# Transcatheter Aortic Valve Replacement for Bicuspid Versus Tricuspid Aortic Stenosis: A Systematic Review and Meta-Analysis

**DOI:** 10.31083/RCM49401

**Published:** 2026-07-28

**Authors:** Fang-Hui Zhu, Xi Peng, Yun-Qi Zhang, Nan Chen, Xiao-Han Zhao, Hui-Ping Zhang

**Affiliations:** ^1^Beijing Hospital, National Center for Gerontology, National Clinical Research Center for Gerontology, The Key Laboratory of Geriatrics of NHC, Institute of Geriatric Medicine, Chinese Academy of Medical Sciences & Peking Union Medical College, 100730 Beijing, China; ^2^Arrhythmia Center, Fuwai Hospital, National Center for Cardiovascular Diseases, Chinese Academy of Medical Sciences, Peking Union Medical College, 100730 Beijing, China

**Keywords:** aortic stenosis, transcatheter aortic valve replacement, bicuspid aortic valve, outcomes, meta-analysis

## Abstract

**Background:**

Although the bicuspid aortic valve (BAV) is a major cause of aortic stenosis (AS), limited evidence exists regarding the safety and efficacy of transcatheter aortic valve replacement (TAVR) in BAV patients. This study aimed to compare TAVR outcomes between BAV and tricuspid aortic valve (TAV) cohorts.

**Methods:**

We conducted a systematic search of PubMed, Web of Science, Embase, and the Cochrane Library to identify studies reporting 1-year follow-up outcomes. Pooled odds ratios (ORs) with 95% confidence intervals (CIs) were derived from a random-effects model.

**Results:**

The analysis included 31 studies involving 192,691 patients. Patients with BAV were younger than those with TAV (71.2 vs. 73.2 years, *p* < 0.01) and exhibited lower society of thoracic surgeons (STS) scores (3.5% vs. 4.3%, *p* < 0.05). Compared with TAV, BAV was associated with a lower likelihood of device success (OR = 0.80; 95% CI 0.65–0.98; *p* = 0.03) and an increased risk of moderate/severe paravalvular leak (PVL) (OR = 1.39; 95% CI 1.22–1.58; *p* < 0.01). No significant differences were observed in in-hospital mortality (OR = 1.10, 95% CI 0.86–1.42; *p* = 0.44), or major peri-procedural complications between the two cohorts. At 30 days, all-cause mortality (OR = 1.19, 95% CI 0.96–1.47, *p* = 0.11) and cardiovascular mortality (OR = 1.59, 95% CI 0.87–2.90; *p* = 0.13) were similar between BAV and TAV groups. While 1-year cardiovascular mortality showed no significant difference (OR = 0.70, 95% CI 0.42–1.16; *p* = 0.17), BAV patients exhibited a significant reduction in 1-year all-cause mortality (OR = 0.84, 95% CI 0.73–0.97; *p* = 0.01). This survival benefit was consistent in propensity-score matched cohorts (OR = 0.78, 95% CI 0.64–0.95; *p* = 0.01). Subgroup analysis further identified distinct survival advantages for BAV patients who were younger (OR = 0.82, 95% CI 0.71–0.95, *p* = 0.01), had lower STS scores (OR = 0.68, 95% CI 0.50–0.92, *p* = 0.01), or received balloon-expandable valves (OR = 0.76, 95% CI 0.61–0.94, *p* = 0.01). Notably, 1-year all-cause mortality rates were similar when stratified by aortic diameter or paravalvular leak (PVL) incidence.

**Conclusion:**

This meta-analysis demonstrated that TAVR had similar safety and efficacy profiles for BAV and TAV patients. Additionally, BAV patients undergoing TAVR exhibited a reduced 1-year all-cause mortality compared to TAV patients.

**The PROSPERO Registration:**

CRD42025618185, https://www.crd.york.ac.uk/PROSPERO/view/CRD42025618185.

## 1. Introduction

Bicuspid aortic valve (BAV), the most common congenital cardiac anomaly, affects approximately 1–2% of the general population [1]. Among these individuals, 12–37% progress to moderate to severe aortic stenosis (AS) [2]. In early transcatheter aortic valve replacement (TAVR) trials, BAV patients were systematically excluded owing to anatomical complexities such as elliptical annuli and asymmetric calcification patterns [3]. Consequently, initial evidence regarding the performance of transcatheter heart valves (THVs) in bicuspid anatomy, as compared with surgical aortic valve replacement (SAVR), remained limited.

Advances in device technology and implantation techniques have established TAVR as a well-validated alternative to SAVR. This therapeutic progress has progressively broadened TAVR eligibility, which now includes patients across the entire surgical risk spectrum [4]. In parallel, growing evidence supporting the feasibility, safety, and efficacy of TAVR in the BAV population has led to its inclusion as a recognized indication for TAVR in recent guidelines, with the highest level of evidence rated as B [4,5,6]. Nonetheless, it is important to note that in these guidelines, the corresponding class of recommendation is typically designated as Class II in most instances, indicating the need for further evidence.

Prior meta-analyses have indicated similar 30-day and 1-year all-cause mortality between BAV and tricuspid aortic valve (TAV) patients undergoing TAVR [3,7,8,9,10]. However, some recent studies have suggested a potential 1-year survival benefit in BAV cohorts, though the underlying explanation remain unclear [6,11,12]. Cardiovascular mortality was rarely evaluated in earlier meta-analyses. Furthermore, beyond basic survival outcomes, effective tools for personalized risk stratification and prognosis prediction after TAVR are still lacking, particularly for complex phenotypes like BAV. Although recent studies have explored machine learning-based models for predicting post-TAVR outcomes, they have not specifically addressed anatomical subtypes such as BAV or provided direct comparisons between BAV and TAV populations [13]. To address this gap in evidence, we conducted a systematic review and meta-analysis to compare peri-procedural, 30-day, and 1-year outcomes between BAV and TAV patients treated with TAVR. By synthesizing the existing data, we aimed to provide a comprehensive assessment of the safety and efficacy of TAVR for severe aortic stenosis in the BAV population.

## 2. Methods

This review was conducted in accordance with Preferred Reporting Items for Systematic Reviews and Meta‐Analyses (PRISMA) guidelines. The protocol was registered with PROSPERO (ID: CRD42025618185); ethical approval was not required.

### 2.1 Data Sources and Search Strategy

A systematic literature search of the PubMed, Web of Science, Embase, and the Cochrane Library databases was conducted to identify studies published up to November 24, 2024. Identified records were managed using EndNote software (Clarivate, Philadelphia, PA, USA), with duplicates removed algorithmically before screening. The detailed search strategy is provided in the **Supplementary Materials**.

### 2.2 Eligibility Criteria

Original studies were included if they met the following criteria: (1) Population: patients undergoing TAVR; (2) Outcome: reported peri-procedural outcomes, and/or 30-day or 1-year mortality; (3) Study Design: randomized controlled trials (RCTs) or observational studies that performed multivariable adjustment for confounders.

Exclusion criteria were: (1) non-human studies or basic science research; (2) non-original research (e.g., case reports, conference abstracts, editorials, reviews without original data); (3) studies evaluating BAV outcomes without a TAV control group; (4) full-text articles published not in English.

**Fig. 1.**
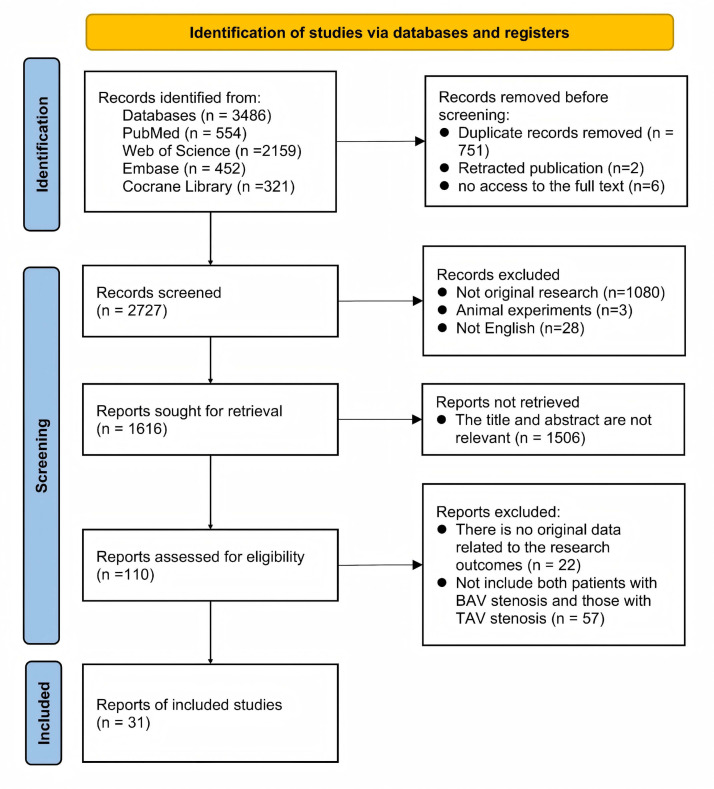
**PRISMA flowchart for inclusion and exclusion of studies from the analysis**. PRISMA, Preferred Reporting Items for Systematic Reviews and Meta-Analyses. BAV, bicuspid aortic valve; TAV, tricuspid aortic valve.

The flow chart of the study is shown (Fig. 1). A total of 3486 records were identified from the initial database search. After removing duplicates and screening the remaining records, 31 studies were ultimately included. Disagreements regarding inclusion were resolved through discussion with a third reviewer until consensus was reached.

### 2.3 Data Extraction

A dual-phase screening process was independently conducted by two investigators. This process included an initial review of titles and abstracts, followed by a full-text assessment of potentially eligible studies.

BAV morphology was classified according to the Sievers classification system into three types: Type 0 (two malformed cusps without a raphe), Type 1 (one raphe), and Type 2 (two raphes) [14]. Our analysis included all three BAV types (0, 1, and 2).

The primary outcome was 1-year mortality, including both all-cause and cardiovascular mortality. Secondary outcomes encompassed 30-day all-cause mortality, 30-day cardiovascular mortality and peri-procedural complications occurring within 30 days or during the hospitalization: device success, life-threatening bleeding, myocardial infarction, coronary obstruction, moderate/severe paravalvular leak (PVL), and major vascular complications. Most of relevant clinical endpoints were defined in accordance with the Valve Academic Research Consortium-3 (VARC-3) criteria except device success [15]. Device success was extracted according to the definitions used in the original studies, which were primarily based on VARC or VARC-2 consensus documents [16,17].

Data were collected including the study name, first author, publication year, study design, number of patients, follow-up duration, baseline characteristics (age, sex, Society of Thoracic Surgeons [STS] score, body mass index [BMI], comorbidities), and outcomes. If values or percentages for outcomes were not explicitly reported, they were calculated from available data. For studies reporting outcomes in propensity-matched populations, results from the matched cohorts were included. Adjusted statistics were used where available.

Data extraction was performed independently by two reviewers using standardized electronic forms. Discrepancies were resolved through consensus, with unresolved disagreements adjudicated by a third senior investigator.

### 2.4 Quality Assessment

The methodological quality of included studies was assessed independently by two investigators using established tools. Cohort studies were appraised using the Newcastle-Ottawa Scale (NOS), which evaluates selection, comparability, and outcome ascertainment on a 9-point scale. Studies with an NOS score below 7 were considered low quality. Notably, NOS scores were utilized for post-inclusion risk-of-bias assessment and sensitivity analysis rather than as a rigid exclusion criterion; this approach was adopted to minimize potential selection bias and ensure a comprehensive synthesis of available evidence. Disagreements were resolved through consensus or arbitration by a third investigator.

### 2.5 Statistical Analysis

Data from all included studies were systematically extracted and aggregated. Categorical variables were summarized as frequencies and percentages (%), and continuous variables as means with standard deviations (SD) for normally distributed data or medians with interquartile ranges (IQR) for non-normally distributed data.

Odds ratios (ORs) were recalculated for all studies based on raw event counts and corresponding sample sizes. Summary effect estimates were derived using an inverse-variance random-effects model with the restricted maximum likelihood (REML) estimator to account for anticipated clinical and methodological heterogeneity across studies. Between-study heterogeneity was assessed using Cochran’s Q test (significance set at *p* < 0.10) and quantified using the *I^2^* statistic (*I^2^* ≤25% = low; 25–50% = moderate; >50% = high heterogeneity).

Potential small-study effects and publication bias were evaluated using funnel plots, supplemented by Egger’s regression test for asymmetry (significance defined as *p* < 0.05). Sensitivity analyses were performed in cases of significant heterogeneity by sequentially excluding each study (‘leave-one-out’ analysis). All analyses were conducted in R (version 4.5.0, R Foundation for Statistical Computing, Vienna, Austria) using the meta package. A two-tailed *p* value < 0.05 was considered statistically significant.

## 3. Results

### 3.1 Study Selection

The study selection process is outlined in the PRISMA flowchart (Fig. 1). A total of 3486 records were identified through the systematic search. After removing duplicates and excluding publications that were reviews, conference abstracts, editorials, or irrelevant to the topic, 110 articles were selected for full-text review. Following a thorough assessment of eligibility, 31 studies were ultimately included in the final analysis [5,18,19,20,21,22,23,24,25,26,27,28,29,30,31,32,33,34,35,36,37,38,39,40,41,42,43,44,45,46,47].

### 3.2 Baseline Characteristics

Baseline characteristics of the included studies and their patient cohorts are summarized in Table 1 (Ref. [5,18,19,20,21,22,23,24,25,26,27,28,29,30,31,32,33,34,35,36,37,38,39,40,41,42,43,44,45,46,47]). The final analysis incorporated a total of 31 studies (15 prospective, 16 retrospective) conducted across multiple countries, including the United States, China, France, Italy, Germany, the UK, Poland, and Hungary, spanning procedures performed between 2005 and 2023. Follow-up durations ranged from 30 days to 3 years, with sample sizes varying from 23 to 170,959 patients, yielding an aggregate cohort of 192,691 TAVR recipients (14,692 [7.6%] with BAV and 177,999 [92.4%] with TAV). Among the 20 studies reporting Sievers morphological subtypes, the distribution among BAV patients was as follows: Type 0 (18.9%), Type 1 (79.2%), and Type 2 (1.9%).

**Table 1.** **Characteristics of **s**tudies **i**ncluded in the **a**nalysis**.

Author	Publication year	Study type	Quality score	Enrollment period	FU	BAV subtype (%)			Total sample size (n)	Event sample size (n)	Mean age (years)	Gender (male%)	STS score (%)	OR/RR/HR	VARC	Adjustment method
						Type 0	Type 1	Type 2								
Costopoulos C et al. [20]	2014	Retrospective Cohort	9/9	2007–2012	1 y	NA	NA	NA	468	21	76.7 ± 7.1	57.0	7.6 ± 4.2	NA	1	NA
Dai H et al. [21]	2023	Retrospective Cohort	8/9	2013–2021	1 y	NA	NA	NA	402	211	74.0 [69.0–79.0]	58.3	4.4 [2.6–7.6]	NA	3	NA
Fu B et al. [5]	2020	Prospective Cohort	9/9	2017–2019	1 y	62.7	11.9	25.4	118	44	73.57 ± 6.30	52.3	6.70 ± 2.95	HR	2	NA
Hong N et al. [22]	2023	Retrospective Cohort	8/9	2017–2021	30 d	NA	NA	NA	389	229	72.9 ± 6.9	65.1	2.6 ± 0.9	NA	NA	NA
Kawamori H et al. [23]	2018	Retrospective Cohort	7/9	2013–2017	3 d	4.9	95.1	0	280	41	80 [70.5–83.0]	68.3	NA	NA	2	NA
Kochman J et al. [24]	2014	Prospective Cohort	8/9	2009–2012	1 y	NA	NA	NA	112	28	77.6 ± 5.5	46.0	NA	NA	1	NA
Liu K et al. [25]	2024	Prospective Cohort	8/9	2018–2019	22 m	NA	NA	NA	65	29	72.1 ± 7.6	65.5	NA	NA	NA	NA
Magyari B et al. [26]	2024	Retrospective Cohort	7/9	2019–2023	30 d	25	73.1	1.9	104	52	71 ± 7.1	65.4	5.2 ± 3.3	NA	2	Propensity‐matched
Makkar RR et al. [27]	2019	Prospective Cohort	8/9	2015–2018	1 y	NA	NA	NA	5382	2691	74 [66–81]	60.4	4.9 [4.0]	HR	2	Propensity‐matched
Makkar RR et al. [28]	2021	Prospective Cohort	8/9	2015–2020	1 y	NA	NA	NA	6336	3168	68.8 ± 8.7	69.2	1.7 [0.6]	HR	NA	Propensity‐matched
Mangieri A et al. [29]	2018	Retrospective Cohort	8/9	2012–2017	30 d	3.7	90.7	1.8	108	54	80 ± 5.3	38.9	4.7 ± 2.7	NA	2	Propensity‐matched
Medranda GA et al. [30]	2021	Prospective Cohort	7/9	NA	1 y	12.8	87.2		107	47	68.4 ± 7.8	44.7	NA	NA	NA	NA
Xiong TY et al. [31]	2018	Retrospective Cohort	8/9	2012–2016	1 y	61.2	38.8	0	116	67	74.0 [68.0–77.0]	59.8	NA	NA	2	NA
Xu Q et al. [32]	2021	Prospective Cohort	7/9	2015–2017	1 y	55.6	44.4	0	23	9	71.2 ± 4.8	33.3	5.5 ± 3.0	NA	2	NA
Zhou D et al. [33]	2022	Retrospective Cohort	8/9	2013–2018	3 y	61.5	36.7	1.8	246	109	75 [71–80]	56.9	5.09 [3.65–8.62]	NA	2	NA
Chodór PA et al. [34]	2021	Retrospective Cohort	9/9	2008–2016	1 y	0	85.7	14.3	83	21	75.76 ±7.96	66.7	NA	NA	2	NA
De Biase C et al. [35]	2018	Prospective Cohort	7/9	2016	30 d	7	93	0	249	83	81.4 ± 7.6	69.0	5.1 ± 3.3	NA	2	NA
Forrest JK et al. [36]	2020	Retrospective Cohort	8/9	2015–2018	1 y	NA	NA	NA	1858	929	73.0 ± 10.3	55.1	NA	NA	1	Multivariable
Kim WK et al. [37]	2021	Retrospective Cohort	7/9	2014–2020	30 d	4.1	95	0.8	968	242	80.0 [75.2–83.5]	62.8	NA	NA	2	NA
Liu XB et al. [38]	2015	Prospective Cohort	8/9	2013–2014	30 d	73.3	26.6	0	40	15	75.4 ± 5.7	60.0	5.6 ± 4.1	NA	1	NA
Michel JM et al. [39]	2021	Prospective Cohort	7/9	2014–2019	1 y	9.0	91	0	743	78	77 [72–81]	61.5	NA	HR	2	NA
Pineda AM et al. [40]	2020	Retrospective Cohort	8/9	2011–2016	2 y	14	86	0	567	50	70 [64–74]	64.0	4.6 [3.0–7.7]	NA	NA	NA
Sannino A et al. [41]	2017	Retrospective Cohort	8/9	2012–2016	1 y	13.6	85.2	1.1	823	88	80.2 ± 8.4	60.2	7.4 ± 3.9	NA	2	NA
Song GY et al. [42]	2018	Prospective Cohort	8/9	2012–2015	2 y	50	38.6	11.4	97	44	73.8 ± 5.2	54.5	5.0 [3.8–8.2]	NA	2	NA
Tung M et al. [43]	2018	Prospective Cohort	7/9	2014–2015	1 y	NA	NA	NA	107	13	74.5 ± 6.44	46.2	NA	NA	2	NA
Yoon SH et al. [44]	2017	Retrospective Cohort	8/9	2005–2016	2 y	12.8	85.6	1.7	1092	546	77.2 ± 8.2	62.8	4.6 ± 4.6	NA	2	Propensity‐matched
Zhou D et al. [45]	2020	Prospective Cohort	7/9	2014–2017	1 y	35.7	64.3	0	110	68	76.41 ± 4.56	45.2	7.42 ± 3.87	NA	2	NA
Deeb GM et al. [46]	2022	Prospective Cohort	8/9	2018–2019	1 y	9.7	90.3	0	290	145	70.5 ± 5.5	53.1	1.4 ± 0.6	NA	3	Propensity‐matched
Williams MR et al. [47]	2022	Prospective Cohort	8/9	NA	1 y	13.6	85.8	0.6	296	148	71.0 [68.0–75.0]	58.1	NA	NA	NA	Propensity‐matched
Arai T et al. [18]	2017	Retrospective Cohort	6/9	2013–2015	30 d	0	90	10	153	10	81.3 ± 5.1	43	NA	NA	2	NA
Halim SA et al. [19]	2020	Retrospective Cohort	5/9	2011–2018	1 y	NA	NA	NA	170,959	5412	74.0 [65.0–81.0]	59.1	NA	NA	2	NA

NA, not available; BAV, bicuspid aortic valve; FU, follow-up; d, days; m, month; y, year; Total Sample Size, the number of bicuspid patients and tricuspid patients; Events Sample Size, the number of bicuspid patients; n, number of patients; STS, society of thoracic surgeons. OR, odds ratio; RR, risk ratio; HR, hazard ratio. VARC, valve academic research consortium.

As shown in **Supplementary Table 1**, BAV patients were significantly younger than those in the TAV cohort (mean age: 71.2 vs. 73.2 years, *p *< 0.01) and had a lower surgical risk profile (mean STS score: 3.5% ± 3.0% vs. 4.3% ± 3.5%, *p* < 0.05). Furthermore, the prevalence of most comorbidities was notably lower in the BAV cohort.

### 3.3 Peri-Procedural Outcomes

**Fig. 2.**
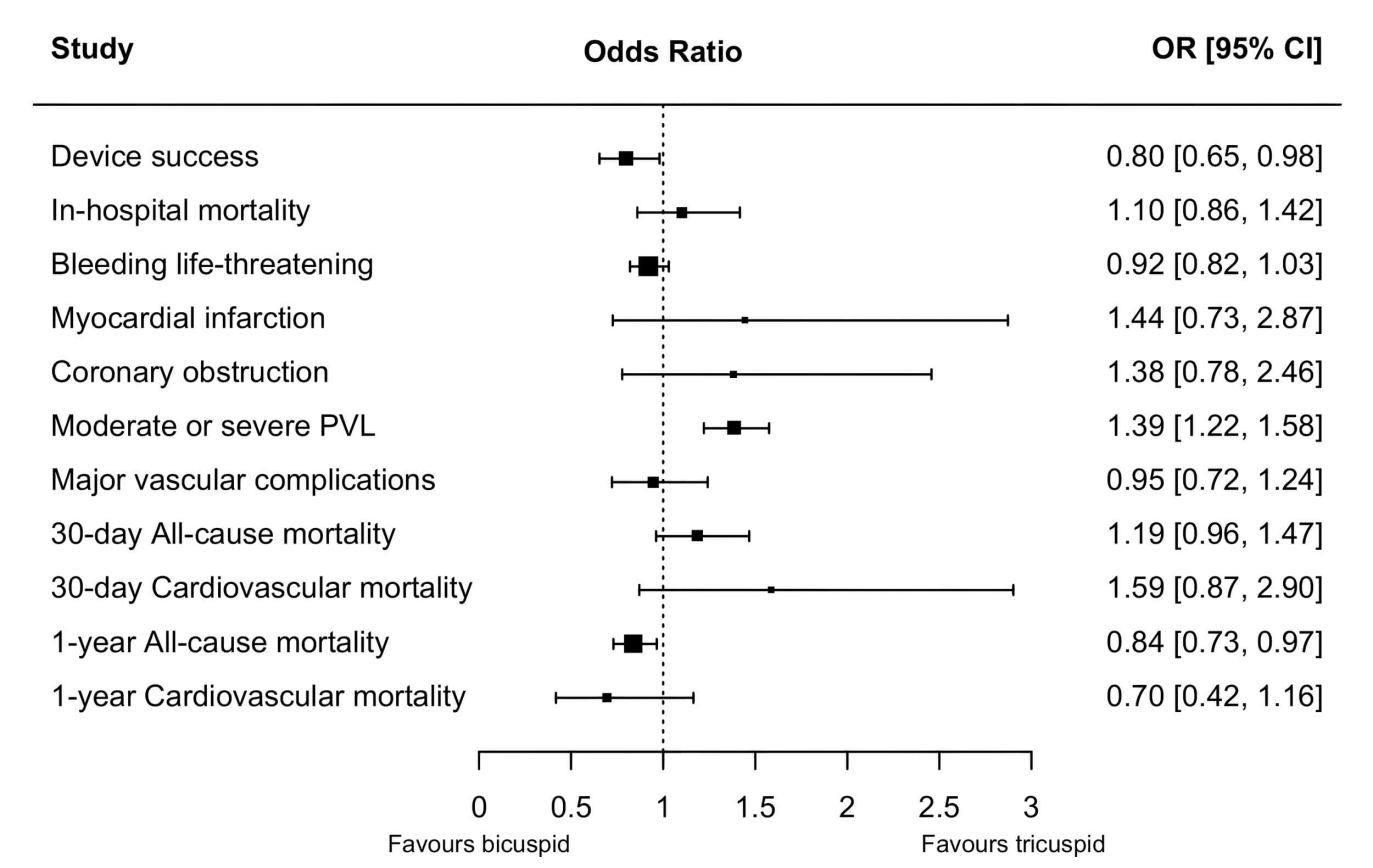
**Forest plot of overall outcomes of this meta‐analysis**. OR, odds ratio; CI, confidence interval; PVL, paravalvular leak.

The distribution of THVs used in the BAV cohort across the included studies was as follows: 26.2% self-expanding (CoreValve, VenusA-valve, Lotus, MicroPort VitaFlow, Evolut R, Evolut PRO, ACURATE neo, Portico, J-valve), 73.5% balloon-expandable (Edwards SAPIEN 3, SAPIEN XT), and 0.1% mechanically expandable. Peri-procedural outcomes are summarized in the **Supplementary Materials**. The BAV cohort exhibited a significantly lower rate of device success (OR = 0.80, 95% CI 0.65–0.98, *p* = 0.03) and a higher incidence of moderate/severe PVL (OR = 1.39, 95% CI 1.22–1.58; *p* < 0.01). Regarding safety profiles, the incidence of in-hospital mortality (OR = 1.10, 95% CI 0.86–1.42; *p* = 0.44), life-threatening bleeding (OR = 0.92, 95% CI 0.82–1.03; *p* = 0.15), myocardial infarction (OR = 1.44, 95% CI 0.73–2.87; *p* = 0.29), coronary obstruction (OR = 1.38, 95% CI 0.78–2.46; *p* = 0.27), and major vascular complications (OR = 0.95, 95% CI 0.72–1.24; *p* = 0.69) showed no statistically significant difference between the two cohorts (Fig. 2).

### 3.4 Clinical Outcomes

**Fig. 3.**
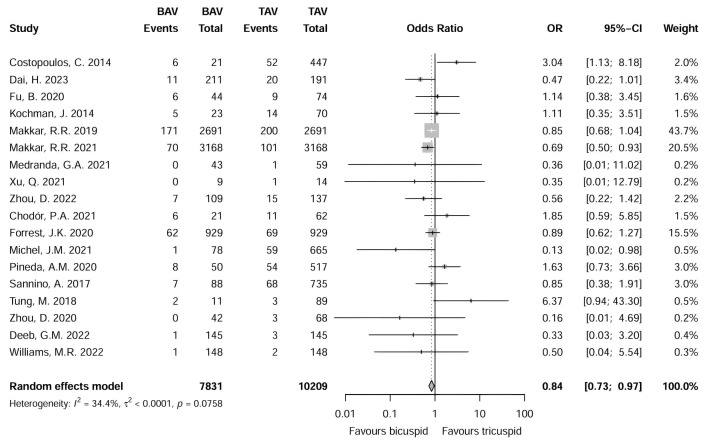
**Forest plot for 1-year all-cause mortality**. OR, odds ratio; CI, confidence interval. *I*^2^, percentage of total variation across studies attributable to heterogeneity rather than chance; τ^2^, between-study variance in a random-effects model; *p* value, Cochran’s Q test for heterogeneity.

At 30 days, no statistically significant differences were observed between the BAV and TAV cohorts regarding all-cause mortality (OR = 1.19, 95% CI 0.96–1.47; *p* = 0.11) or cardiovascular mortality (OR = 1.59, 95% CI 0.87–2.90; *p* = 0.13). However, at 1-year follow-up, the BAV group exhibited a significantly lower risk of all-cause mortality compared to the TAV group (OR = 0.84, 95% CI 0.73–0.97; *p* = 0.01) (Fig. 3), while cardiovascular mortality remained similar between the two groups (OR = 0.70, 95% CI 0.42–1.16; *p* = 0.17).

**Fig. 4.**
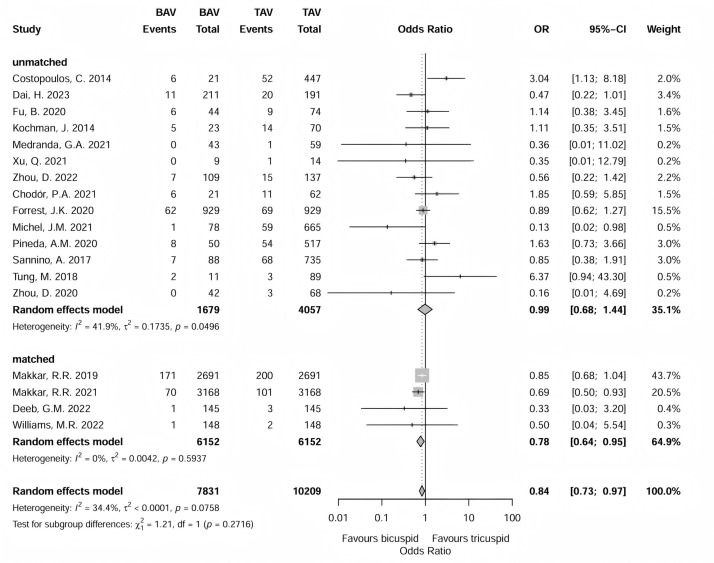
**Forest plot for 1-year all-cause mortality of propensity-score matched cohorts**. OR, odds ratio; CI, confidence interval. Matched, propensity-score matched cohort. *I^2^*, percentage of total variation across studies attributable to heterogeneity rather than chance; τ^2^, between-study variance in a random-effects model; *p* value, Cochran’s Q test for heterogeneity.

In analyses restricted to propensity-score matched cohorts, the BAV group demonstrated a further reduction in the risk of 1-year all-cause mortality relative to the TAV group (OR = 0.78, 95% CI 0.64–0.95; *p* = 0.01) (Fig. 4). All pooled estimates indicated minimal between-study heterogeneity (*I^2^* <50%).

### 3.5 Subgroup Analysis

**Fig. 5.**
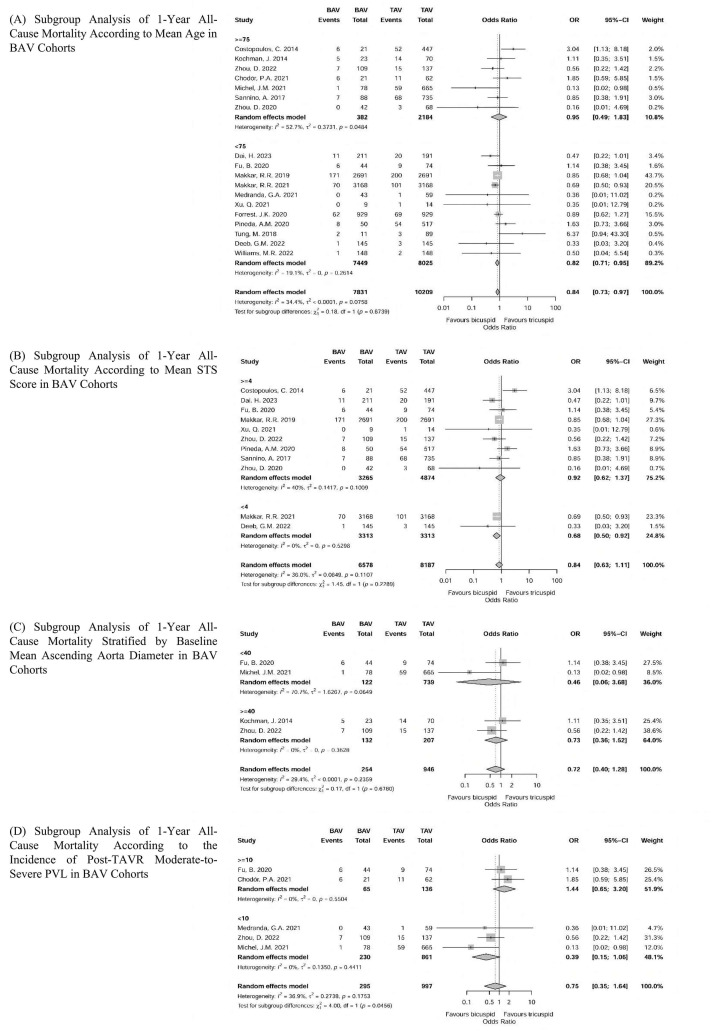
**Subgroup analyses of 1-year all-cause mortality by mean age (A), mean STS score (B), mean ascending aorta diameter (C), and moderate/severe PVL (D)**. OR, odds ratio; CI, confidence interval. STS, Society of Thoracic Surgeons; PVL, paravalvular leak. *I^2^*, percentage of total variation across studies attributable to heterogeneity rather than chance; τ^2^, between-study variance in a random-effects model; *p* value, Cochran’s Q test for heterogeneity.

In the subgroup analysis at 1-year follow-up, patients with BAV under 75 years of mean age exhibited a lower risk of all-cause mortality compared to patients with TAV (OR = 0.82, 95% CI 0.71–0.95, *p* = 0.01, *I^2^* = 0%); whereas no mortality difference was observed in those aged 75 years or older (OR = 0.95, 95% CI 0.49–1.83, *p* = 0.87) (Fig. 5). Similarly, within the low-risk cohort defined by an STS score <4%, the BAV group demonstrated a superior survival profile relative to the TAV group (OR = 0.68, 95% CI 0.50–0.92, *p* = 0.01, *I^2^* = 0%). However, this mortality advantage was not replicated in the higher-risk subgroup (STS score ≥4%), where outcomes were comparable between the two groups (OR = 0.92, 95% CI 0.62–1.37, *p* = 0.69, *I^2^* = 40%).

**Fig. 6.**
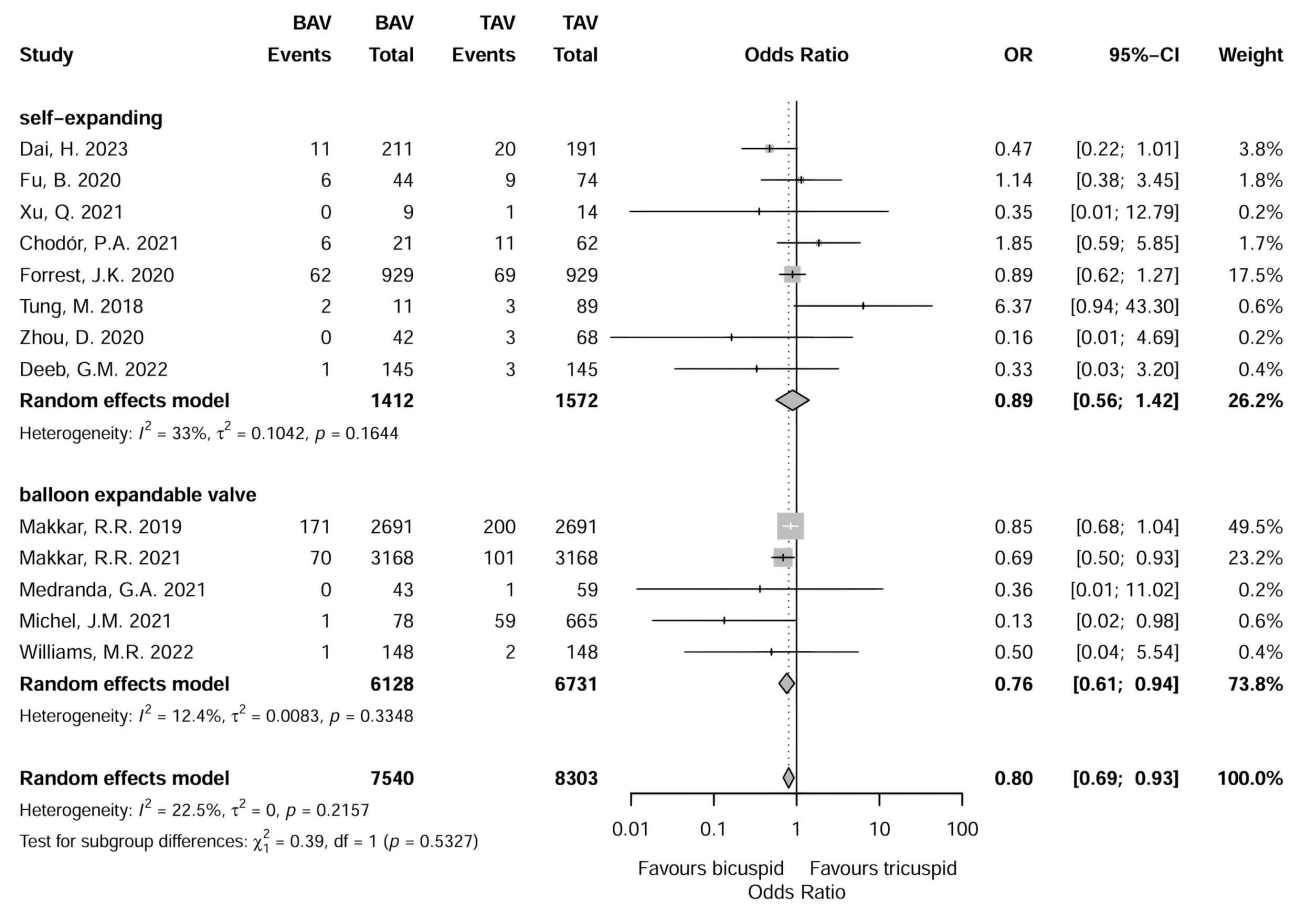
**Subgroup analysis of valve type in 1-year all-cause mortality**. OR, odds ratio; CI, confidence interval.

In analyses stratified by baseline mean ascending aorta diameter of BAV cohorts, for mean ascending aortic diameter <40 mm (OR = 0.46, 95% CI 0.06–3.68, *I^2^* = 70.7%) nor the subgroup with diameter ≥40 mm (OR = 0.73, 95% CI 0.36–1.52, *p* = 0.40, *I^2^* = 0%) (Fig. 5) showed a statistically significant association. When comparing the outcomes based on the transcatheter valve type, analyses of self-expanding valves indicated no significant difference in 1-year mortality between BAV and TAV patients (OR = 0.89, 95% CI 0.56–1.42, *p* = 0.63, *I^2^* = 33%) (Fig. 6). Conversely, among patients treated with balloon-expandable valves, those with BAV exhibited a superior 1-year survival rate compared to TAV patients (OR = 0.76, 95% CI 0.61–0.94, *p* = 0.01, *I^2^* = 12.4%). The BAV cohort was categorized into two subgroups based on a 10% threshold of moderate-to-severe PVL incidence post-TAVR. In both the <10% (OR = 0.39, 95% CI 0.15–1.06, *p* = 0.07, *I^2^* = 0%) and ≥10% (OR = 1.44, 95% CI 0.65–3.20, *p* = 0.37, *I^2^* = 0%) subgroups, no significant differences in 1-year all-cause mortality were observed between the BAV and TAVR groups.

### 3.6 Bias Evaluation

The risk of bias and sensitivity analyses are detailed in the **Supplementary Materials**. Funnel plot assessments indicated minimal publication bias, with most outcomes showing negligible heterogeneity. Moderate statistical heterogeneity was observed for device success (*I^2^* = 42.3%, *p*_Heterogeneity_ < 0.05). Sensitivity analyses confirmed robustness for most clinical endpoints (e.g., mortality, bleeding), while subgroup analyses revealed variable heterogeneity depending on patient characteristics (age, STS score) and valve type. Sensitivity analysis excluding low-quality studies (NOS <7) yielded results consistent with the primary analysis, further confirming the robustness of our findings (**Supplementary Fig. 4**).

## 4. Discussion

The objective of our analysis was to assess the therapeutic safety and efficacy of TAVR in AS patients with BAV versus TAV, focusing on peri-procedural, 30-day and 1-year outcomes. The main findings of our analysis were as follows: (1) Compared with TAV patients, BAV patients were significantly younger and exhibited a lower surgical risk profile, as well as a reduced prevalence of comorbidities. (2) Peri-procedural outcomes were statistically comparable, which confirmed equivalent therapeutic safety and efficacy of TAVR in patients with BAV and TAV. (3) The BAV cohort exhibited a significantly lower risk of 1-year all-cause mortality. (4) Subgroup analysis revealed a survival benefit in BAV patients with younger age, lower STS scores, or those who received balloon-expandable valves. In contrast, 1-year all-cause mortality rates were similar between BAV and TAV patients when stratified by aortic diameter or PVL severity.

Our analysis demonstrated no significant differences between BAV and TAV patients in life-threatening bleeding, myocardial infarction, coronary obstruction, in-hospital mortality, or major vascular complications. These results suggested that TAVR had comparable safety and efficacy profiles for both valve types, indicating that valve morphology is not a primary determinant of procedural outcomes in contemporary TAVR practice. This aligned with prior studies, such as those by Makkar et al [27], which reported similar rates of procedure complications or in-hospital events between BAV and TAV patients. However, patients with BAV, relative to those with TAV, demonstrated a decreased probability of achieving device success alongside a significantly higher incidence of moderate/severe PVL, which necessitate further investigation into patient-specific procedural planning and the refinement of device-sizing strategies.

In this study, the comparable 30-day all-cause mortality between BAV and TAV patients aligned with the findings of most recent meta-analyses [3,11,48]. An exception was the meta-analysis by Gupta et al. (2023) [10], which reported marginally higher 30-day all-cause mortality in BAV patients; however, this was not observed in their subgroup analysis restricted to new-generation devices. As for 30-day cardiovascular mortality, there was no significant difference between the BAV and TAV cohorts, which was consistent with the prior studies reporting no significant intergroup differences [3,10].

This analysis revealed a significantly lower 1-year all-cause mortality risk in BAV patients following TAVR. This aligns with recent studies showing a survival advantage for BAV patients at one year [6,11,12], in contrast to earlier reports with comparable outcomes between BAV and TAV cohorts [3,8,9]. Our meta-analysis incorporates a more comprehensive and updated literature search, including pivotal studies published within recent years, which may account for this shift in observed outcomes. The survival benefit appeared primarily due to the more favorable baseline characteristics in BAV patients, rather than procedural factors or valve morphology. Specifically, the BAV cohort was younger on average and had a lower surgical risk profile, both identified as independent predictors of mid-term mortality following TAVR in previous studies [49,50]. Subgroup analyses further supported this, showing a mortality benefit mainly in patients under 75 years. Generally, BAV patients were younger, with fewer comorbidities, and had potentially more resilient cardiac anatomy. Fan et al. [51] identified age and left ventricular pressure after valve release as independent predictors of long-term mortality in BAV patients. Older patients often have poorer nutritional status and a higher incidence of comorbidities, which may increase long-term mortality risk after the procedure. Thus, younger age likely contributed to the survival advantage. Additionally, a subgroup analysis based on the STS score showed that BAV patients with an STS score <4% had a lower risk of 1-year all-cause mortality compared to TAV patients. These findings corroborate those reported by Al-Asad et al. [6], who observed reduced 1 year mortality in the BAV group among low surgical risk patients, further supporting the safety profile of TAVR in the BAV population. Beyond age and STS score, Zghouzi et al. [12] attributed the better 1-year survival in BAV patients to their lower comorbidity burden. Our analysis showed a statistically lower prevalence of multiple comorbidities in the BAV group, which may help explain their survival advantage.

However, a propensity-matched analysis by Al-Asad et al. [6], which balanced baseline characteristics between groups, still identified lower 1-year mortality in the BAV cohort. Our finding was similarly corroborated by the analysis. A meta-analysis of propensity-score matched cohorts demonstrated that the BAV population had a significant survival advantage over the TAV population in terms of 1-year all-cause mortality. This suggests that factors beyond baseline characteristics, such as age and comorbidities, may also influence long-term outcomes.

In addition, BAV was frequently associated with aortopathies, including dilation of the ascending or descending aorta. These anatomical abnormalities can elevate the risk of stroke during the manipulation of major vessels. He et al. reported that baseline ascending aortic diameter predicted 1‑year mortality after TAVR in BAV but not in TAV patients, and was the sole predictor of mortality in those with type 0 morphology [52]. Accordingly, we performed a subgroup analysis stratified by ascending aortic diameter. The analysis revealed no significant difference in 1-year mortality between the comparison groups for either the <40 mm or the ≥40 mm subgroups. Nevertheless, the prognostic role of ascending aortic dilation in BAV patients merits further long‑term follow‑up.

PVL, a common complication after TAVR, often requires further treatment when symptoms reach moderate/severe levels. Kodali et al. identified PVL as a predictor of 1-year mortality in aortic stenosis patients undergoing TAVR [53]. Our analysis showed a non-significant trend toward lower mortality in BAV patients with moderate/severe PVL. The impact of PVL on the survival advantage of BAV patients requires further investigation.

Finally, our analysis revealed no significant intergroup difference in 1-year cardiovascular mortality. The dissociation between reduced all-cause mortality and neutral cardiovascular mortality implies that the survival advantage in BAV patients may be largely attributable to a lower incidence of non-cardiac death. Kolte et al., analyzing data from the STS/ACC TVT Registry, found that approximately two-thirds of deaths within 1-year post-TAVR were from noncardiac causes, with pulmonary conditions, infections, and neurologic events (including stroke) constituting the most frequent categories [54]. This supports the view that the 1-year survival benefit in BAV patients may be influenced predominantly by non-cardiovascular factors.

## 5. Limitations

Our analysis had some limitations. First, a number of the studies included in our meta-analysis did not report specific outcomes in early vs. newer generation devices; therefore, subgroup analysis comparing complications and outcomes between different devices in BAV and TAV patients could not be performed. Second, most of the studies included did not report outcomes within subtypes of BAV, or they lacked complete data on the bicuspid subtypes. Subsequently, we could not compare outcomes within BAV subtypes to explore whether different BAV subtypes show prognostic differences after TAVR. Third, given that the predominant body of evidence in this review lacked propensity score matching to address inherent baseline disparities, our pooled analysis was based on the limited subset of studies that provided adjusted estimates through matching techniques. Fourth, although ORs were used as the primary effect measure due to data availability and reporting patterns, their limitations should be acknowledged. ORs may overestimate effect sizes when outcomes are common and may be influenced by between-study heterogeneity. These factors should be considered when interpreting the results. Fifth, the NOS used for quality assessment has inherent limitations, including limited inter-rater reliability and a focus on study reporting rather than deep methodological rigor. The scale may not fully account for the nuances of statistical adjustments, such as propensity score matching versus multivariable regression. While our sensitivity analyses support the robustness of the findings, the potential for residual confounding in the included observational studies remains a factor in our interpretation. Lastly, only nonrandomized observational studies were included in this analysis, as no RCTs met the eligibility criteria, thereby restricting the generalizability of the results. Nonetheless, we rigorously assessed observational study quality and performed design-based subgroup analyses. The consistency of risk factor effects across study types strengthens the overall reliability of our findings.

## 6. Conclusion

This meta-analysis indicates that TAVR exhibits comparable safety and efficacy in both BAV and TAV populations. Moreover, patients with BAV undergoing TAVR demonstrate a more favorable prognosis compared to those with TAV. Our study underscored a significantly improved survival in BAV patients who were younger, had lower STS scores, and received balloon-expandable valves.

## Data Availability

The raw data extracted from included studies, data used for all analyses, analytic code, and other materials used in the systematic review are available upon request from the corresponding author.

## Abbreviations

BAV, bicuspid aortic valve; TAV, tricuspid aortic valve; TAVR, transcatheter aortic valve replacement; SAVR, surgical aortic valve replacement; AS, aortic stenosis; STS, society of thoracic surgeons; BMI, body mass index; CI, confidence Interval; NOS, Newcastle‐Ottawa Scale; OR, odds ratio; PRISMA, Preferred Reporting Items for Systematic Reviews and Meta‐Analyses; VARC, valve academic research consortium; PVL, paravalvular leak.
