# Supplementary material for: Transcatheter Aortic Valve Replacement for Bicuspid Versus Tricuspid Aortic Stenosis: A Systematic Review and Meta-Analysis
Source: Rev Cardiovasc Med. 2026 Jul 28;27(7):49401. doi: 10.31083/RCM49401 (PMC13419958; doi:10.31083/RCM49401)

Supplementary Materials

Supplementary Method 1. Database sources, search strategy and search results.

Supplementary Table 1. Baseline characteristics of the included patients.

Supplementary Table 2. Device success rates and corresponding VARC definitions across included studies.

Supplementary Figure 1. Funnel plots for primary and secondary endpoints.

Supplementary Figure 2. Forest plots for primary and secondary endpoints.

Supplementary Figure 3. Sensitivity analyses for primary and secondary endpoints: 'leave-one-out'.

Supplementary Figure 4. Sensitivity analyses of clinical endpoints excluding studies with a Newcastle-Ottawa Scale (NOS) score <7.

Supplementary Method 1

Database search results

Database	Search strategy	Articles
**PubMed**	(((("Transcatheter Aortic Valve Replacement"[Mesh]) OR (Transcatheter Aortic Valve Replacement[Title/Abstract])) OR (Transcatheter Aortic Valve Implantation[Title/Abstract])) AND ((((((((((((((("Bicuspid Aortic Valve Disease"[Mesh]) OR (Bicuspid Aortic Valve Disease[Title/Abstract])) OR (Bicuspid Aortic Valve[Title/Abstract])) OR (Bicuspid Aortic Valves[Title/Abstract])) OR (Valve, Bicuspid Aortic[Title/Abstract])) OR (Aortic Valve, Bicuspid[Title/Abstract])) OR (Aortic Valve Disease 1[Title/Abstract])) OR (Two-Raphe Bicuspid Aortic Valve[Title/Abstract])) OR (Bicuspid Aortic Valve, Two-Raphe[Title/Abstract])) OR (Bicuspid Aortic Valve, Two Raphe[Title/Abstract])) OR (Double Raphe Bicuspid Aortic Valve[Title/Abstract])) OR (Two Raphe Bicuspid Aortic Valve[Title/Abstract])) OR (Single Raphe Bicuspid Aortic Valve[Title/Abstract])) OR (Bicuspid Aortic Valve, Single Raphe[Title/Abstract])) OR (Purely Bicuspid Aortic Valve[Title/Abstract]))) AND (((((((((("Aortic Valve Stenosis"[Mesh]) OR (Aortic Valve Stenosis[Title/Abstract])) OR (Aortic Valve Stenoses[Title/Abstract])) OR (Stenoses, Aortic Valve[Title/Abstract])) OR (Stenosis, Aortic Valve[Title/Abstract])) OR (Valve Stenoses, Aortic[Title/Abstract])) OR (Valve Stenosis, Aortic[Title/Abstract])) OR (Aortic Stenosis[Title/Abstract])) OR (Stenoses, Aortic[Title/Abstract])) OR (Stenosis, Aortic[Title/Abstract]))	**554**
**Web of Science**	(TS=(Transcatheter Aortic Valve Replacement) OR AB=(Transcatheter Aortic Valve Replacement OR Transcatheter Aortic Valve Implantation)) AND (TS=(Bicuspid Aortic Valve Disease) OR AB=(Bicuspid	**2159**

	Aortic Valve Disease OR Bicuspid Aortic Valve OR Bicuspid Aortic Valves OR Valve, Bicuspid Aortic OR Aortic Valve, Bicuspid OR Aortic Valve Disease 1 OR Two-Raphe Bicuspid Aortic Valve OR Bicuspid Aortic Valve, Two-Raphe OR Bicuspid Aortic Valve, Two Raphe OR Double Raphe Bicuspid Aortic Valve OR Two Raphe Bicuspid Aortic Valve OR Single Raphe Bicuspid Aortic Valve OR Bicuspid Aortic Valve, Single Raphe OR Purely Bicuspid Aortic Valve)) AND (TS=(Aortic Valve Stenosis) OR AB=(Aortic Valve Stenosis OR Aortic Valve Stenoses OR Stenoses, Aortic Valve OR Stenosis, Aortic Valve OR Valve Stenoses, Aortic OR Valve Stenosis, Aortic OR Aortic Stenosis OR Stenoses, Aortic OR Stenosis, Aortic))	
**Embace**	#1<br>Transcatheter Aortic Valve Replacement OR Transcatheter Aortic Valve Implantation<br>#2<br>Bicuspid Aortic Valve Disease OR Bicuspid Aortic Valve OR Bicuspid Aortic Valves OR Valve, Bicuspid Aortic OR Aortic Valve, Bicuspid OR Aortic Valve Disease 1 OR Two-Raphe Bicuspid Aortic Valve OR Bicuspid Aortic Valve, Two-Raphe OR Bicuspid Aortic Valve, Two Raphe OR Double Raphe Bicuspid Aortic Valve OR Two Raphe Bicuspid Aortic Valve OR Single Raphe Bicuspid Aortic Valve OR Bicuspid Aortic Valve, Single Raphe OR Purely Bicuspid Aortic Valve<br>#3<br>Aortic Valve Stenosis OR Aortic Valve Stenoses OR Stenoses, Aortic Valve OR Stenosis, Aortic Valve OR Valve Stenoses, Aortic OR Valve Stenosis, Aortic OR Aortic Stenosis OR Stenoses, Aortic OR Stenosis, Aortic<br>#1 AND #2 AND #3	**452**
**Cocrane library**	#1<br>Bicuspid Aortic Valve Disease or Bicuspid Aortic Valve, Two Raphe or Bicuspid Aortic Valve, Two-Raphe or Two-Raphe Bicuspid Aortic Valve or Double Raphe Bicuspid Aortic Valve or Two Raphe Bicuspid Aortic Valve or Single Raphe Bicuspid Aortic Valve or Bicuspid Aortic Valve, Single Raphe or	**321**

	Purely Bicuspid Aortic Valve or Bicuspid Aortic Valve or Bicuspid Aortic Valves or Aortic Valve, Bicuspid or Valve, Bicuspid Aortic or Aortic Valve Disease<br>#2<br>Aortic Valve Stenosis or Stenoses, Aortic Valve or Valve Stenosis, Aortic or Stenosis, Aortic Valve or Stenoses, Aortic or Stenosis, Aortic or Aortic Valve Stenoses or Valve Stenoses, Aortic or Aortic Stenosis<br>#3<br>Transcatheter Aortic Valve Replacement or Transcatheter Aortic Valve Implantation<br>#1 and #2 and #3

Supplementary Table 1

Supplementary Table 1. Baseline characteristics of the included patients. Statistically significant differences were marked with an asterisk (*).

Baseline variable	BAV	TAV	P value
Age (years, mean ± SD) *	71.2±9.2 (14692)	73.2±10 (177999)	<0.001
Men (%) *	61.3(9009/14689)	53.3(94930/177963)	<0.001
STS score (%, mean ± SD) *	3.5±3.0(7627)	4.3±3.5(9217)	0.048
BMI (kg/m2, mean ± SD)	28.9±6.9(7087)	28.5±6.2(9498)	0.11
Hypertension (%) *	78.1(7155/9156)	80.8 (9964/12336)	0.016
Diabetes (%)	29.5(2702/9166)	30.7(3790/12331)	0.307
CAD (%) *	47.6(2915/6127)	61.4(102950/167730)	<0.001
COPD (%)	22.5(197/876)	25.4(851/3346)	0.088
CKD (%) *	26.9(205/762)	34.6(658/1903)	<0.001
NYHA classification (III/IV) (%) *	70.2(9774/13930)	76.2(132759/174114)	0.02
prior PCI (%) *	19.3(537/2789)	25(1032/4123)	0.017
Prior MI (%) *	4.9(64/1298)	12.9(378/2941)	<0.001

BAV=bicuspid aortic valve; TAV=tricuspid aortic valve; STS=society of thoracic surgeons; BMI=Body mass index; CAD= coronary artery disease; COPD= chronic obstructive pulmonary disease; CKD=chronic kidney disease; PCI=percutaneous coronary intervention.

Supplementary Table 2

Supplementary Table 2. Device success rates and corresponding VARC definitions across included studies.

Author	Year	Device success-BAV	Total-BAV	Device success-TAV	Total-TAV	VARC version
Costopoulos, C.	2014	18	21	422	447	VARC1
Dai, H.	2023	174	211	157	191	VARC2
Fu, B.	2020	38	44	66	74	VARC2
Kawamori, H.	2018	40	41	232	239	VARC2
Kochman, J.	2014	26	28	78	84	VARC1
Magyari, B.	2024	52	52	52	52	VARC2
Makkar, R.R.	2019	2577	2671	2586	2678	VARC2
Makkar, R.R.	2021	3044	3156	3066	3152	NA
De Biase, C.	2018	61	83	155	166	NA
Forrest, J.K.	2020	893	929	887	929	VARC1
Kim, W.K.	2021	105	242	331	726	NA
Liu, X.B.	2015	13	15	22	25	VARC1
Michel, J.M.	2021	73	78	600	665	NA
Yoon, S.H.	2017	466	546	499	546	NA
Deeb, G.M.	2022	137	143	136	141	VARC1
Halim, S. A.	2020	5146	5361	158959	164369	NA

BAV=bicuspid aortic valve; TAV=tricuspid aortic valve; VARC: Valve Academic Research Consortium; NA=not available.

Supplementary Figure 1

Supplementary Figure 1. Funnel plots for primary and secondary endpoints.

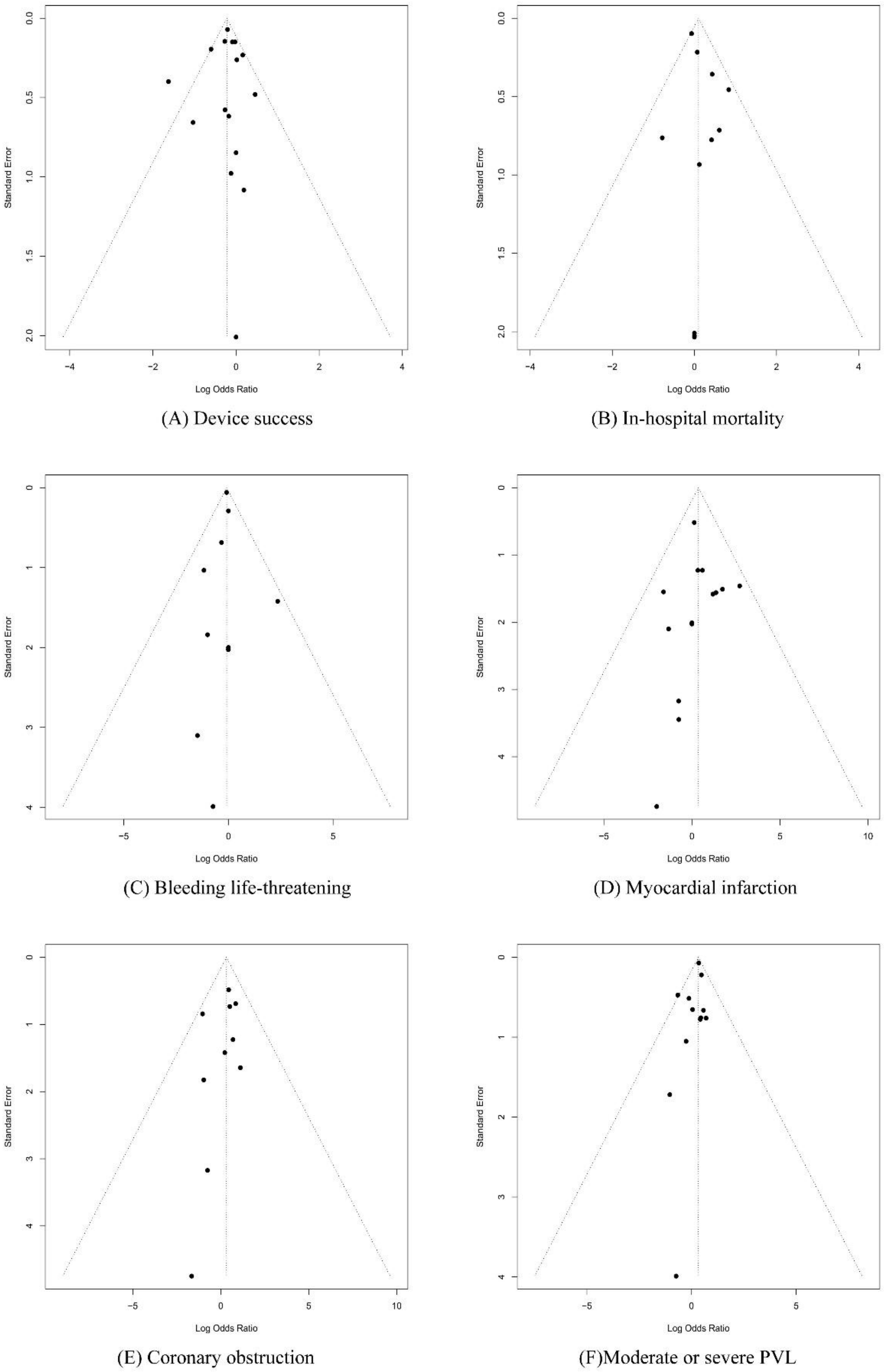

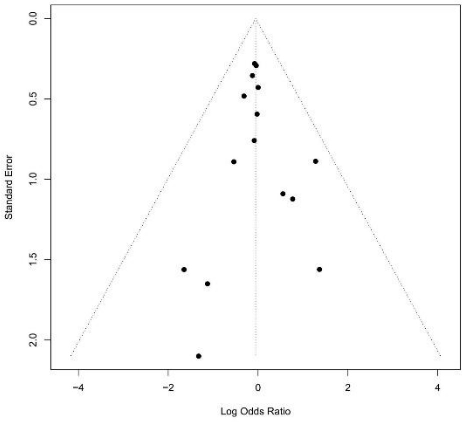

(G) Major vascular complications

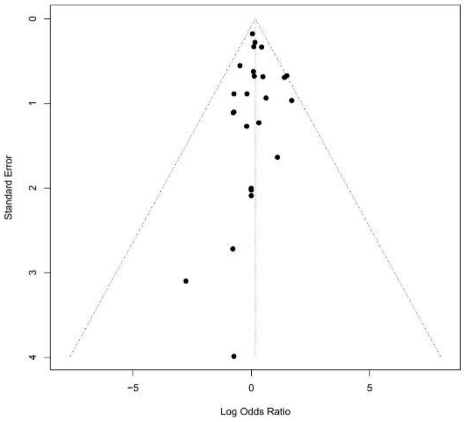

(H) 30-day All-cause mortality

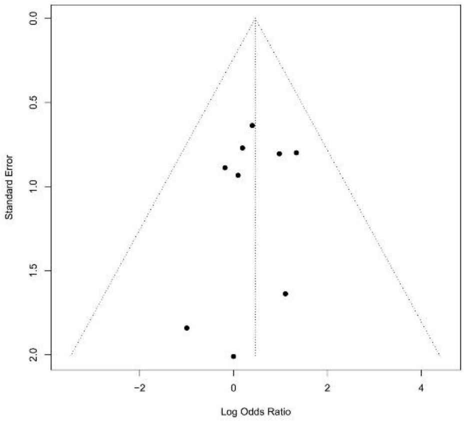

(I) 30-day Cardiovascular mortality

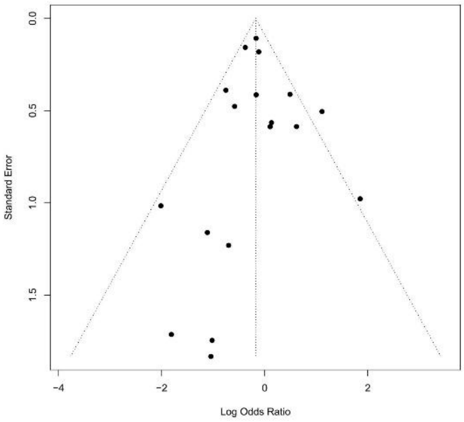

(J) 1-year All-cause mortality

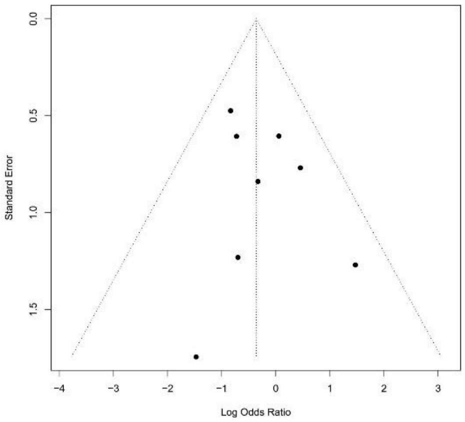

(K) 1-year Cardiovascular mortality

Supplementary Figure 2

Supplementary Figure 2. Forest plots for primary and secondary endpoints.

(A) Forest Plot for Device success OR

Study	BAV Events	BAV Total	TAV Events	TAV Total	OR	95%−CI	Weight
Costopoulos, C. 2014	18	21	422	447	0.36	[0.10; 1.29]	2.2%
Dai, H. 2023	174	211	157	191	1.02	[0.61; 1.70]	8.1%
Fu, B. 2020	38	44	66	74	0.77	[0.25; 2.38]	2.7%
Kawamori, H. 2018	40	41	232	239	1.21	[0.14; 10.07]	0.9%
Kochman, J. 2014	26	28	78	84	1.00	[0.19; 5.26]	1.4%
Magyari, B. 2024	52	52	52	52	1.00	[0.02; 51.34]	0.3%
Makkar, R.R. 2019	2577	2671	2586	2678	0.98	[0.73; 1.31]	12.5%
Makkar, R.R. 2021	3044	3156	3066	3152	0.76	[0.57; 1.01]	12.6%
De Biase, C. 2018	61	83	155	166	0.20	[0.09; 0.43]	4.8%
Forrest, J.K. 2020	893	929	887	929	1.17	[0.75; 1.85]	9.1%
Kim, W.K. 2021	105	242	331	726	0.91	[0.68; 1.23]	12.4%
Liu, X.B. 2015	13	15	22	25	0.89	[0.13; 6.02]	1.0%
Michel, J.M. 2021	73	78	600	665	1.58	[0.62; 4.06]	3.6%
Yoon, S.H. 2017	466	546	499	546	0.55	[0.37; 0.80]	10.5%
Deeb, G.M. 2022	137	143	136	141	0.84	[0.25; 2.82]	2.4%
Halim, S. A. 2020	5146	5361	158959	164369	0.81	[0.71; 0.94]	15.6%
**Random effects model**		**13621**		**174484**	**0.80**	**[0.65; 0.98]**	**100.0%**

Heterogeneity: $I^2 = 42.3\%$, $\tau^2 = 0.0632$, $p = 0.0382$

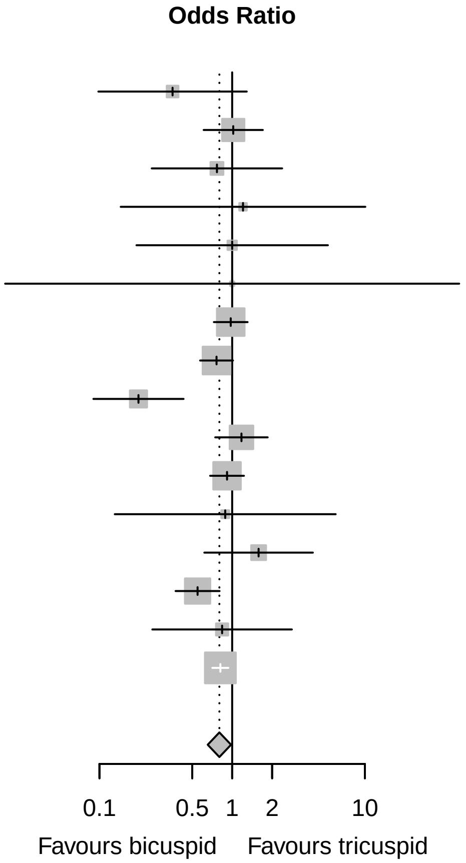

(B) Forest Plot for In-hospital mortality OR

Study	BAV Events	BAV Total	TAV Events	TAV Total	OR	95%−CI	Weight
Dai, H. 2023	6	211	3	191	1.83	[0.45; 7.44]	3.0%
Fu, B. 2020	2	44	3	74	1.13	[0.18; 7.02]	1.8%
Hong, N. 2023	0	229	0	160	1.00	[0.02; 53.96]	0.4%
Magyari, B. 2024	0	52	0	52	1.00	[0.02; 51.34]	0.4%
Makkar, R.R. 2019	45	2691	42	2691	1.07	[0.70; 1.64]	22.9%
Makkar, R.R. 2021	20	3168	13	3168	1.54	[0.77; 3.10]	10.7%
Medranda, G.A. 2021	0	47	0	60	1.00	[0.02; 52.85]	0.4%
De Biase, C. 2018	3	83	4	166	1.52	[0.33; 6.95]	2.6%
Forrest, J.K. 2020	16	929	7	929	2.31	[0.95; 5.64]	7.0%
Kim, W.K. 2021	2	242	13	726	0.46	[0.10; 2.04]	2.7%
Halim, S. A. 2020	110	5412	3598	165543	0.93	[0.77; 1.13]	48.1%
**Random effects model**		**13108**		**173760**	**1.10**	**[0.86; 1.42]**	**100.0%**

Heterogeneity: $I^2 = 0.0\%$, $\tau^2 = 0.0242$, $p = 0.6799$

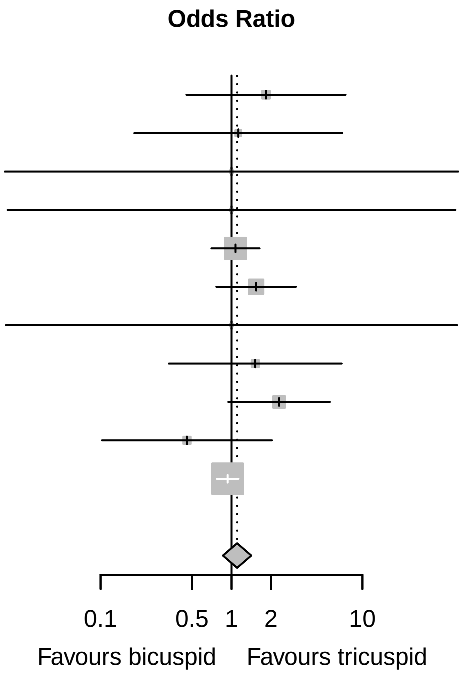

(C) Forest Plot for Bleeding life-threatening OR

Study	BAV Events	BAV Total	TAV Events	TAV Total	OR	95%−CI	Weight
Costopoulos, C. 2014	1	21	62	447	0.31	[0.04; 2.36]	0.3%
Kochman, J. 2014	3	28	12	84	0.72	[0.19; 2.76]	0.7%
Liu, K. 2024	0	29	0	36	1.00	[0.02; 53.12]	0.1%
Makkar, R.R. 2019	0	2691	0	2691	1.00	[0.02; 50.42]	0.1%
Makkar, R.R. 2021	0	3168	0	3168	1.00	[0.02; 50.41]	0.1%
Kim, W.K. 2021	17	242	51	726	1.00	[0.57; 1.77]	4.0%
Liu, X.B. 2015	0	15	1	25	0.37	[0.01; 13.63]	0.1%
Pineda, A.M. 2020	1	50	1	517	10.53	[0.65; 170.98]	0.2%
Sannino, A. 2017	0	88	3	735	0.23	[0.00; 99.81]	0.0%
Song, G.Y. 2018	0	44	0	53	1.00	[0.02; 52.30]	0.1%
Arai, T. 2017	0	10	1	143	0.48	[0.00; 1195.76]	0.0%
Halim, S. A. 2020	303	5412	10042	165547	0.92	[0.82; 1.03]	94.3%
**Random effects model**		**11798**		**174172**	**0.92**	**[0.82; 1.03]**	**100.0%**

Heterogeneity: $I^2 = 0.0\%$, $\tau^2 = 0$, $p = 0.9434$

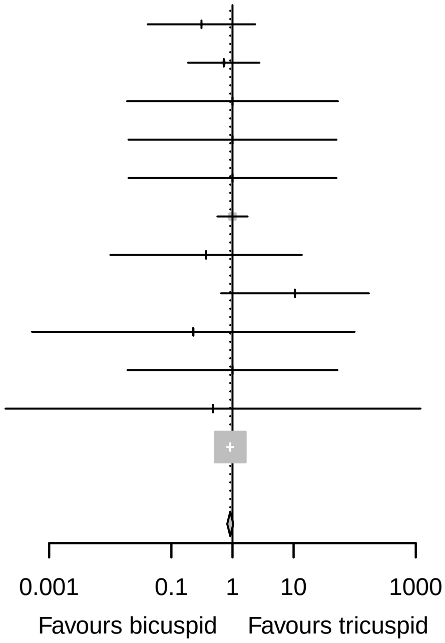

(D) Forest Plot for Myocardial infarction OR

Study	BAV Events	BAV Total	TAV Events	TAV Total	OR	95%−CI	Weight
Costopoulos, C. 2014	0	21	6	447	0.14	[0.00; 1471.10]	0.5%
Dai, H. 2023	2	211	1	191	1.82	[0.16; 20.21]	8.1%
Hong, N. 2023	2	229	1	160	1.40	[0.13; 15.58]	8.1%
Kochman, J. 2014	0	28	2	84	0.27	[0.00; 16.33]	2.8%
Liu, K. 2024	0	29	0	36	1.00	[0.02; 53.12]	3.0%
Magyari, B. 2024	0	52	0	52	1.00	[0.02; 51.34]	3.0%
Makkar, R.R. 2019	8	2691	7	2691	1.14	[0.41; 3.16]	45.8%
Makkar, R.R. 2021	7	3168	0	3168	15.03	[0.86; 263.33]	5.8%
Zhou, D. 2022	1	109	0	137	3.29	[0.15; 73.12]	4.9%
Forrest, J.K. 2020	0	929	2	929	0.20	[0.01; 4.16]	5.1%
Liu, X.B. 2015	1	15	0	25	3.92	[0.18; 83.55]	5.1%
Michel, J.M. 2021	0	78	1	665	0.47	[0.00; 236.81]	1.2%
Pineda, A.M. 2020	0	50	1	517	0.48	[0.00; 409.36]	1.0%
Song, G.Y. 2018	2	44	0	53	5.66	[0.29; 109.24]	5.4%
**Random effects model**		**7654**		**9155**	**1.44**	**[0.73; 2.87]**	**100.0%**

Heterogeneity: $I^2 = 0.0\%$, $\tau^2 = 0$, $p = 0.8952$

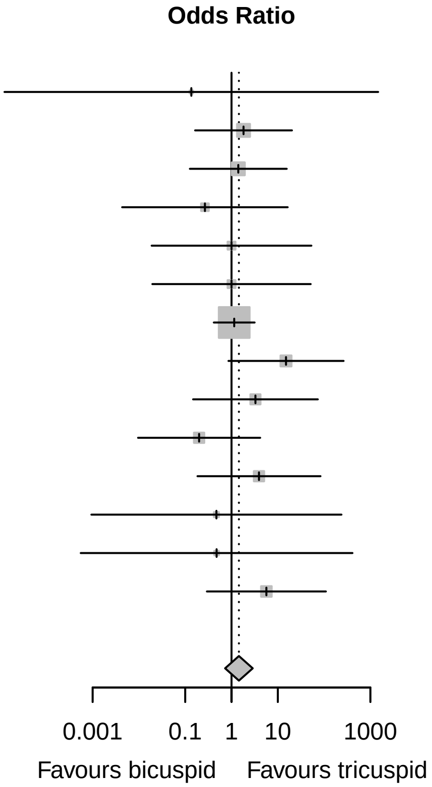

(E) Forest Plot for Coronary obstruction OR

Study	BAV Events	BAV Total	TAV Events	TAV Total	Odds Ratio	OR	95%−CI	Weight
Costopoulos, C. 2014	0	21	4	447		0.19	[0.00; 2099.23]	0.4%
Dai, H. 2023	2	211	5	191		0.36	[0.07; 1.86]	12.1%
Fu, B. 2020	0	44	1	74		0.38	[0.01; 13.61]	2.6%
Makkar, R.R. 2019	11	2689	7	2683		1.57	[0.61; 4.06]	36.7%
Makkar, R.R. 2021	7	3168	3	3168		2.34	[0.60; 9.04]	18.1%
Mangieri, A. 2018	1	54	0	54		3.06	[0.12; 76.70]	3.2%
Zhou, D. 2022	1	109	1	137		1.26	[0.08; 20.37]	4.3%
Forrest, J.K. 2020	2	929	1	929		2.00	[0.18; 22.12]	5.7%
Michel, J.M. 2021	0	78	1	665		0.47	[0.00; 236.81]	0.9%
Yoon, S.H. 2017	5	546	3	546		1.67	[0.40; 7.03]	16.0%
**Random effects model**		**7849**		**8894**		**1.38**	**[0.78; 2.46]**	**100.0%**

Heterogeneity: $I^2 = 0.0\%$, $\tau^2 = 0$, $p = 0.8814$

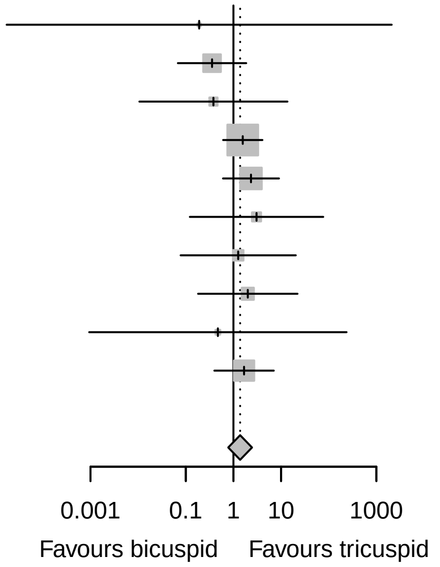

(F) Forest Plot for Moderate/severe PVL OR

Study	BAV Events	BAV Total	TAV Events	TAV Total	OR	95%−CI	Weight
Fu, B. 2020	5	44	5	74	1.77	[0.48; 6.49]	1.0%
Hong, N. 2023	6	229	4	160	1.05	[0.29; 3.78]	1.0%
Medranda, G.A. 2021	0	47	1	60	0.35	[0.01; 10.27]	0.1%
Zhou, D. 2022	7	109	16	137	0.52	[0.21; 1.31]	1.9%
Chodór, P.A. 2021	3	21	6	62	1.56	[0.35; 6.86]	0.7%
De Biase, C. 2018	3	83	4	166	1.52	[0.33; 6.95]	0.7%
Kim, W.K. 2021	5	241	17	726	0.88	[0.32; 2.42]	1.6%
Michel, J.M. 2021	1	78	11	665	0.77	[0.10; 6.06]	0.4%
Song, G.Y. 2018	5	44	3	50	2.01	[0.45; 8.94]	0.7%
Yoon, S.H. 2017	57	546	37	546	1.60	[1.04; 2.47]	8.7%
Arai, T. 2017	0	10	1	143	0.48	[0.00; 1195.76]	0.0%
Halim, S. A. 2020	215	4856	4753	149442	1.41	[1.23; 1.62]	83.1%
**Random effects model**		**6308**		**152231**	**1.39**	**[1.22; 1.58]**	**100.0%**

Heterogeneity: $I^2 = 0.0\%$, $\tau^2 = 0$, $p = 0.7839$

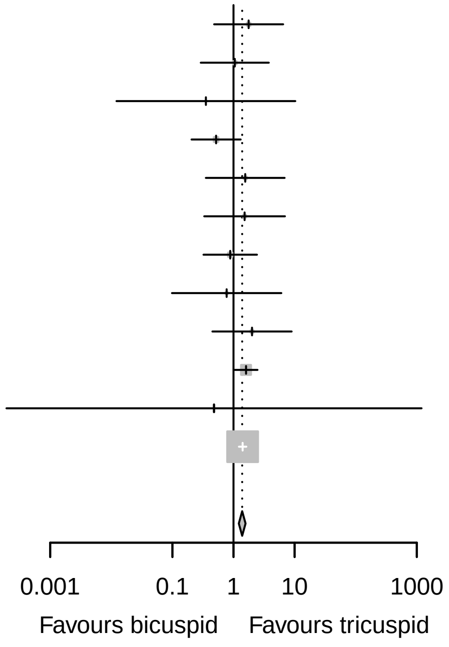

(G) Forest Plot for Major vascular complications OR

Study	BAV Events	BAV Total	TAV Events	TAV Total	OR	95%−CI	Weight
Costopoulos, C. 2014	2	21	46	447	0.92	[0.21; 4.07]	3.3%
Fu, B. 2020	4	44	2	74	3.60	[0.63; 20.53]	2.4%
Hong, N. 2023	7	229	5	160	0.98	[0.30; 3.14]	5.4%
Kochman, J. 2014	0	28	2	84	0.27	[0.00; 16.33]	0.4%
Liu, K. 2024	0	36	1	36	0.32	[0.01; 8.23]	0.7%
Magyari, B. 2024	0	52	2	52	0.19	[0.01; 4.11]	0.8%
Makkar, R.R. 2019	23	2691	24	2691	0.96	[0.54; 1.70]	22.3%
Makkar, R.R. 2021	15	3168	17	3168	0.88	[0.44; 1.77]	15.2%
Forrest, J.K. 2020	11	929	11	929	1.00	[0.43; 2.32]	10.4%
Kim, W.K. 2021	18	242	58	726	0.93	[0.53; 1.60]	24.4%
Liu, X.B. 2015	1	15	0	25	3.92	[0.18; 83.55]	0.8%
Michel, J.M. 2021	5	78	57	665	0.73	[0.28; 1.88]	8.2%
Pineda, A.M. 2020	1	50	6	517	1.74	[0.21; 14.73]	1.6%
Song, G.Y. 2018	2	44	4	53	0.58	[0.10; 3.35]	2.4%
Arai, T. 2017	1	10	7	143	2.16	[0.24; 19.51]	1.5%
**Random effects model**		**7637**		**9770**	**0.95**	**[0.72; 1.24]**	**100.0%**

Heterogeneity: $I^2 = 0.0\%$, $\tau^2 = 0$, $p = 0.9548$

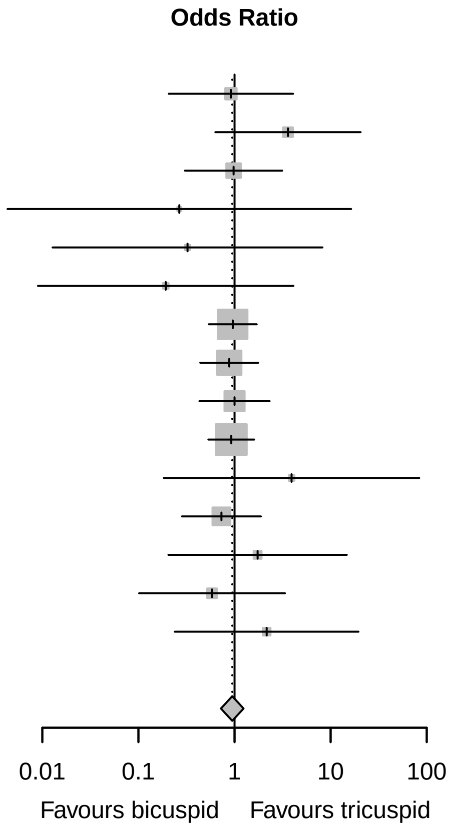

(H) Forest Plot for 30-day All−cause mortality OR

Study	BAV Events	BAV Total	TAV Events	TAV Total	OR	95%−CI	Weight
Costopoulos, C. 2014	3	21	16	447	4.49	[1.20; 16.81]	2.6%
Dai, H. 2023	5	211	4	191	1.13	[0.30; 4.29]	2.5%
Fu, B. 2020	2	44	4	74	0.83	[0.15; 4.75]	1.5%
Hong, N. 2023	2	213	1	146	1.37	[0.12; 15.30]	0.8%
Kawamori, H. 2018	0	41	1	239	0.46	[0.00; 94.73]	0.2%
Kochman, J. 2014	1	28	6	84	0.48	[0.06; 4.18]	1.0%
Magyari, B. 2024	0	52	0	52	1.00	[0.02; 51.34]	0.3%
Makkar, R.R. 2019	66	2691	63	2691	1.05	[0.74; 1.49]	36.6%
Makkar, R.R. 2021	28	3168	24	3168	1.17	[0.68; 2.02]	14.9%
Mangieri, A. 2018	2	54	4	54	0.48	[0.08; 2.74]	1.5%
Medranda, G.A. 2021	0	47	0	60	1.00	[0.02; 52.85]	0.3%
Xu, Q. 2021	0	9	0	14	1.00	[0.02; 60.27]	0.3%
Chodór, P.A. 2021	1	21	6	62	0.47	[0.05; 4.12]	0.9%
De Biase, C. 2018	4	83	5	166	1.63	[0.43; 6.24]	2.5%
Forrest, J.K. 2020	23	929	15	929	1.55	[0.80; 2.98]	10.3%
Kim, W.K. 2021	4	242	19	720	0.62	[0.21; 1.84]	3.8%
Liu, X.B. 2015	1	15	2	25	0.82	[0.07; 9.91]	0.7%
Michel, J.M. 2021	0	78	13	665	0.06	[0.00; 27.50]	0.1%
Pineda, A.M. 2020	3	50	8	517	4.06	[1.04; 15.83]	2.4%
Sannino, A. 2017	3	88	23	735	1.09	[0.32; 3.72]	3.0%
Song, G.Y. 2018	3	44	2	53	1.87	[0.30; 11.70]	1.3%
Tung, M. 2018	2	13	3	94	5.52	[0.83; 36.71]	1.2%
Yoon, S.H. 2017	20	546	18	546	1.12	[0.58; 2.13]	10.6%
Deeb, G.M. 2022	1	145	0	145	3.02	[0.12; 74.77]	0.4%
Williams, M.R. 2022	0	148	0	148	1.00	[0.02; 50.73]	0.3%
Arai, T. 2017	0	10	1	143	0.48	[0.00; 1195.76]	0.1%
**Random effects model**		**8991**		**12168**	**1.19**	**[0.96; 1.47]**	**100.0%**

Heterogeneity: $I^2 = 0.0\%$, $\tau^2 = 0$, $p = 0.8945$

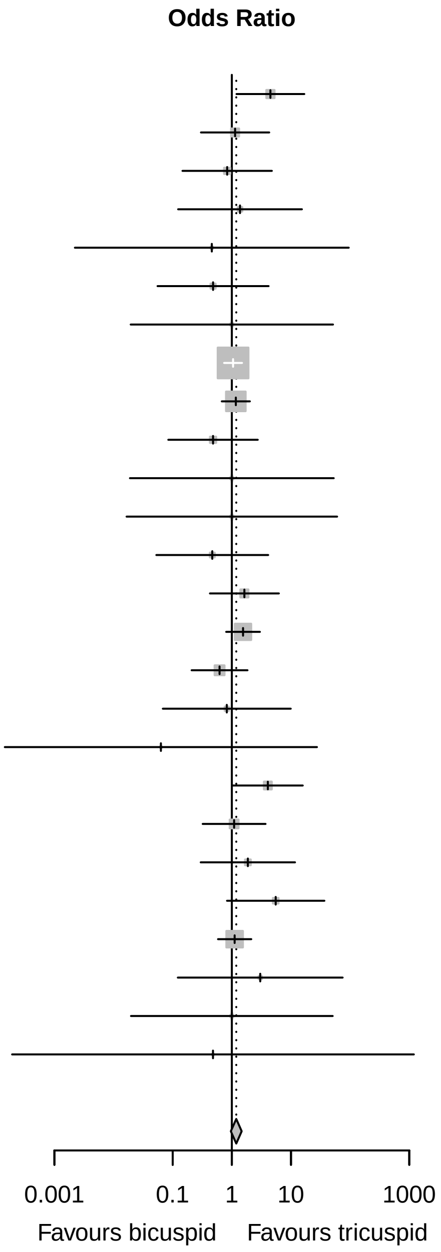

(I) Forest Plot for 30-day Cardiovascular mortality OR

Study	BAV Events	BAV Total	TAV Events	TAV Total	Odds Ratio	OR	95%−CI	Weight
Costopoulos, C. 2014	2	21	12	447		3.82	[0.80; 18.27]	14.8%
Dai, H. 2023	4	211	3	191		1.21	[0.27; 5.48]	15.9%
Fu, B. 2020	2	44	4	74		0.83	[0.15; 4.75]	12.0%
Magyari, B. 2024	0	52	0	52		1.00	[0.02; 51.34]	2.3%
Xiong, T.Y. 2018	3	67	2	49		1.10	[0.18; 6.86]	10.8%
Liu, X.B. 2015	0	15	1	25		0.37	[0.01; 13.63]	2.8%
Pineda, A.M. 2020	2	50	8	517		2.65	[0.55; 12.84]	14.6%
Sannino, A. 2017	3	88	17	735		1.49	[0.43; 5.19]	23.3%
Deeb, G.M. 2022	1	145	0	145		3.02	[0.12; 74.77]	3.5%
**Random effects model**		**693**		**2235**		**1.59**	**[0.87; 2.90]**	**100.0%**

Heterogeneity: $I^2 = 0.0\%$, $\tau^2 = 0$, $p = 0.9171$

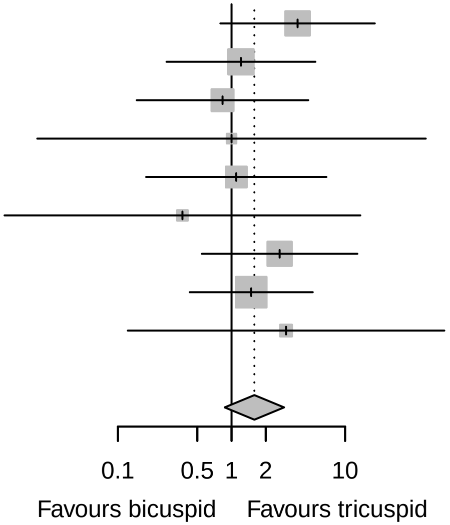

(J) Forest Plot for 1−year All−cause mortality OR

Study	BAV Events	BAV Total	TAV Events	TAV Total	OR	95%−CI	Weight
Costopoulos, C. 2014	6	21	52	447	3.04	[1.13; 8.18]	2.0%
Dai, H. 2023	11	211	20	191	0.47	[0.22; 1.01]	3.4%
Fu, B. 2020	6	44	9	74	1.14	[0.38; 3.45]	1.6%
Kochman, J. 2014	5	23	14	70	1.11	[0.35; 3.51]	1.5%
Makkar, R.R. 2019	171	2691	200	2691	0.85	[0.68; 1.04]	43.7%
Makkar, R.R. 2021	70	3168	101	3168	0.69	[0.50; 0.93]	20.5%
Medranda, G.A. 2021	0	43	1	59	0.36	[0.01; 11.02]	0.2%
Xu, Q. 2021	0	9	1	14	0.35	[0.01; 12.79]	0.2%
Zhou, D. 2022	7	109	15	137	0.56	[0.22; 1.42]	2.2%
Chodór, P.A. 2021	6	21	11	62	1.85	[0.59; 5.85]	1.5%
Forrest, J.K. 2020	62	929	69	929	0.89	[0.62; 1.27]	15.5%
Michel, J.M. 2021	1	78	59	665	0.13	[0.02; 0.98]	0.5%
Pineda, A.M. 2020	8	50	54	517	1.63	[0.73; 3.66]	3.0%
Sannino, A. 2017	7	88	68	735	0.85	[0.38; 1.91]	3.0%
Tung, M. 2018	2	11	3	89	6.37	[0.94; 43.30]	0.5%
Zhou, D. 2020	0	42	3	68	0.16	[0.01; 4.69]	0.2%
Deeb, G.M. 2022	1	145	3	145	0.33	[0.03; 3.20]	0.4%
Williams, M.R. 2022	1	148	2	148	0.50	[0.04; 5.54]	0.3%
**Random effects model**		**7831**		**10209**	**0.84**	**[0.73; 0.97]**	**100.0%**

Heterogeneity: $I^2 = 34.4\%$, $\tau^2 < 0.0001$, $p = 0.0758$

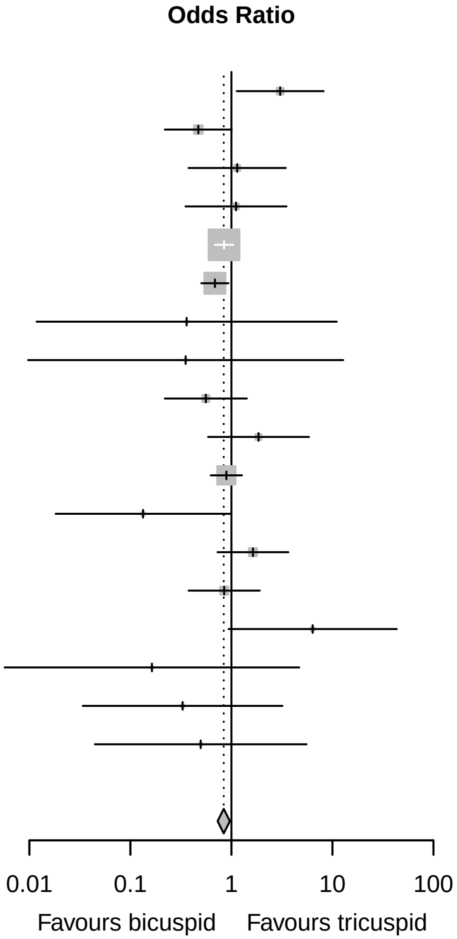

(K) Forest Plot for 1−year Cardiovascular mortality OR

Study	BAV Events	BAV Total	TAV Events	TAV Total	Odds Ratio	OR	95%−CI	Weight
Costopoulos, C. 2014	2	21	28	447		1.58	[0.35; 7.10]	11.6%
Dai, H. 2023	7	211	14	191		0.43	[0.17; 1.10]	30.4%
Fu, B. 2020	5	44	8	74		1.06	[0.32; 3.46]	18.7%
Xiong, T.Y. 2018	3	67	3	49		0.72	[0.14; 3.72]	9.7%
Zhou, D. 2022	4	109	10	137		0.48	[0.15; 1.59]	18.6%
Tung, M. 2018	1	11	2	89		4.35	[0.36; 52.37]	4.2%
Zhou, D. 2020	0	42	2	68		0.23	[0.01; 6.99]	2.2%
Deeb, G.M. 2022	1	145	2	145		0.50	[0.04; 5.54]	4.5%
**Random effects model**		**650**		**1200**		**0.70**	**[0.42; 1.16]**	**100.0%**

Heterogeneity: $I^2 = 0.0\%$, $\tau^2 = 0$, $p = 0.5958$

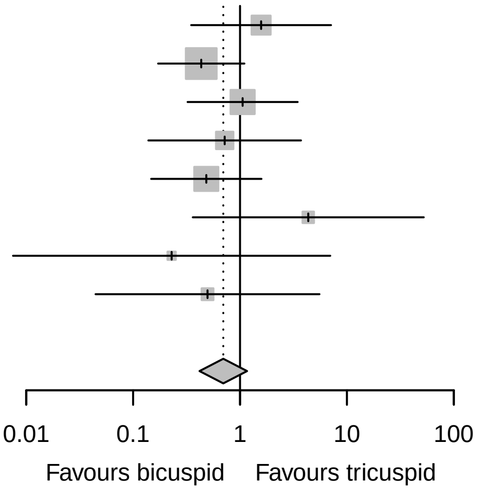

Supplementary Figure 3

Supplementary Figure 3. Sensitivity analyses for primary and secondary endpoints: 'leave-one-out'.

(A) Sensitivity Analysis for Device success

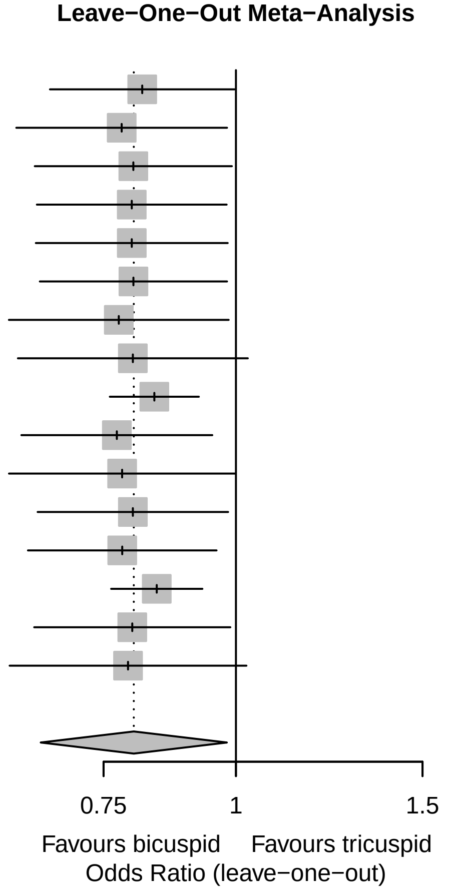

Study omitted	OR	95%−CI	P−value	Tau2	Tau	I2
Omitting Costopoulos, C. 2014	0.82	[0.67; 1.00]	0.0466	0.0594	0.2437	42.5%
Omitting Dai, H. 2023	0.78	[0.62; 0.98]	0.0335	0.0818	0.2860	44.6%
Omitting Fu, B. 2020	0.80	[0.65; 0.99]	0.0413	0.0726	0.2694	46.1%
Omitting Kawamori, H. 2018	0.80	[0.65; 0.98]	0.0314	0.0664	0.2576	45.8%
Omitting Kochman, J. 2014	0.80	[0.65; 0.98]	0.0333	0.0682	0.2611	46.0%
Omitting Magyari, B. 2024	0.80	[0.65; 0.98]	0.0318	0.0642	0.2533	46.1%
Omitting Makkar, R.R. 2019	0.78	[0.61; 0.98]	0.0367	0.0876	0.2960	42.8%
Omitting Makkar, R.R. 2021	0.80	[0.62; 1.03]	0.0788	0.1009	0.3176	45.5%
Omitting De Biase, C. 2018	0.84	[0.76; 0.92]	0.0003	0	0	0%
Omitting Forrest, J.K. 2020	0.77	[0.63; 0.95]	0.0145	0.0588	0.2425	40.3%
Omitting Kim, W.K. 2021	0.78	[0.61; 1.00]	0.0487	0.0962	0.3102	44.8%
Omitting Liu, X.B. 2015	0.80	[0.65; 0.98]	0.0339	0.0669	0.2587	46.1%
Omitting Michel, J.M. 2021	0.78	[0.64; 0.96]	0.0181	0.0622	0.2493	41.9%
Omitting Yoon, S.H. 2017	0.84	[0.76; 0.93]	0.0007	0	0	34.7%
Omitting Deeb, G.M. 2022	0.80	[0.65; 0.99]	0.0383	0.0719	0.2681	46.1%
Omitting Halim, S. A. 2020	0.79	[0.61; 1.02]	0.0740	0.1074	0.3277	46.1%
**Random effects model**	**0.80**	**[0.65; 0.98]**	**0.0315**	**0.0632**	**0.2514**	**42.3%**

(B) Sensitivity Analysis for In-hospital mortality

Study omitted	OR	95%−CI	P−value	Tau2	Tau	I2
Omitting Dai, H. 2023	1.08	[0.85; 1.37]	0.5489	0.0198	0.1408	0%
Omitting Fu, B. 2020	1.11	[0.86; 1.43]	0.4333	0.0267	0.1635	0%
Omitting Hong, N. 2023	1.11	[0.86; 1.42]	0.4350	0.0249	0.1578	0%
Omitting Magyari, B. 2024	1.11	[0.86; 1.42]	0.4349	0.0249	0.1578	0%
Omitting Makkar, R.R. 2019	1.19	[0.83; 1.72]	0.3509	0.0664	0.2576	0%
Omitting Makkar, R.R. 2021	1.02	[0.83; 1.26]	0.8423	0.0077	0.0876	0%
Omitting Medranda, G.A. 2021	1.11	[0.86; 1.42]	0.4350	0.0249	0.1578	0%
Omitting De Biase, C. 2018	1.09	[0.85; 1.41]	0.4883	0.0237	0.1539	0%
Omitting Forrest, J.K. 2020	0.99	[0.84; 1.17]	0.8934	0	0	0%
Omitting Kim, W.K. 2021	1.16	[0.88; 1.53]	0.2989	0.0356	0.1887	0%
Omitting Halim, S. A. 2020	1.26	[0.93; 1.71]	0.1350	0	0	0%
**Random effects model**	**1.10**	**[0.86; 1.42]**	**0.4395**	**0.0242**	**0.1555**	**0%**

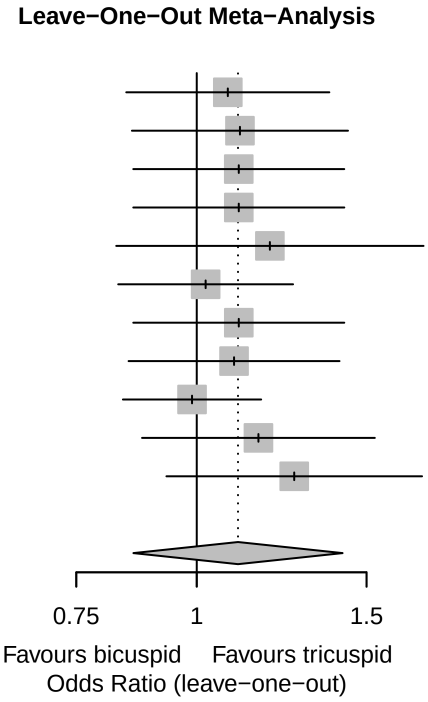

(C) Sensitivity Analysis for Bleeding life-threatening

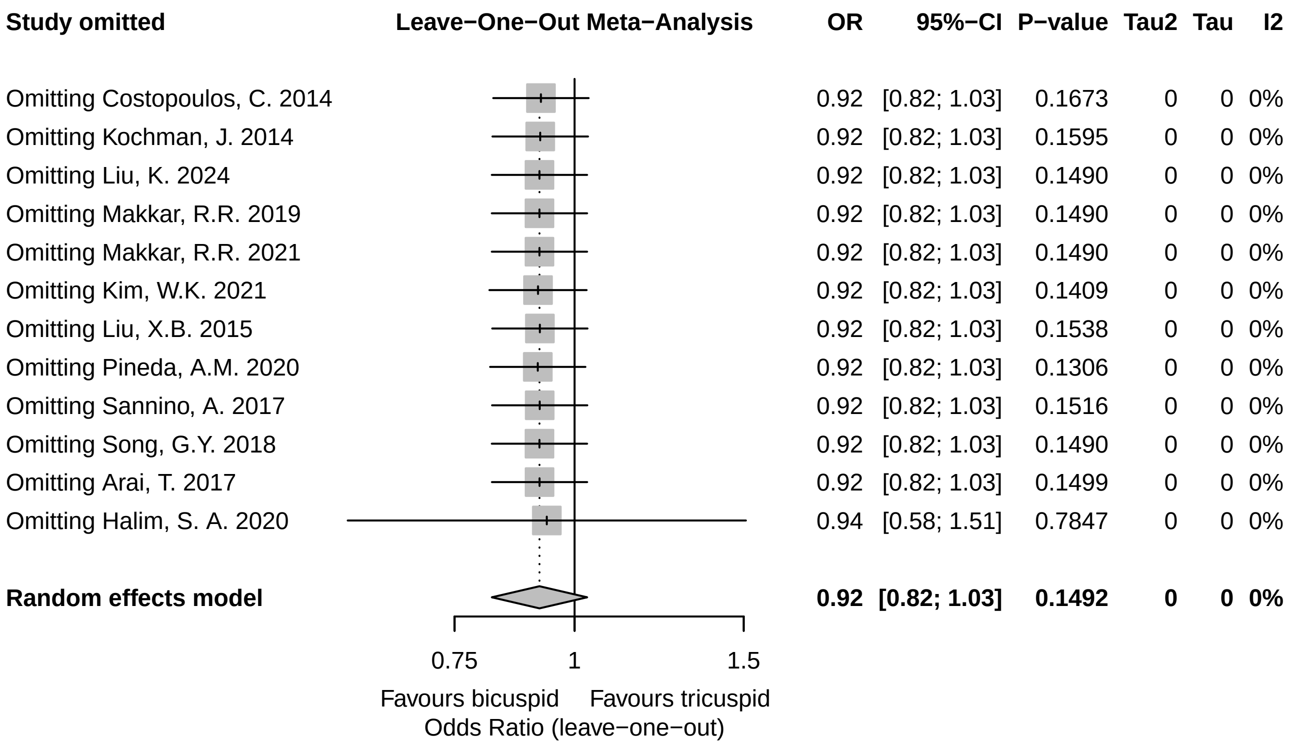

(D) Sensitivity Analysis for Myocardial infarction

Study omitted	OR	95%−CI	P−value	Tau2	Tau	I2
Omitting Costopoulos, C. 2014	1.46	[0.73; 2.92]	0.2792	0	0	0%
Omitting Dai, H. 2023	1.42	[0.69; 2.90]	0.3429	0	0	0%
Omitting Hong, N. 2023	1.45	[0.71; 2.97]	0.3117	0	0	0%
Omitting Kochman, J. 2014	1.52	[0.75; 3.04]	0.2422	0	0	0%
Omitting Liu, K. 2024	1.46	[0.73; 2.94]	0.2873	0	0	0%
Omitting Magyari, B. 2024	1.46	[0.73; 2.94]	0.2872	0	0	0%
Omitting Makkar, R.R. 2019	1.76	[0.69; 4.48]	0.2356	0	0	0%
Omitting Makkar, R.R. 2021	1.25	[0.62; 2.54]	0.5349	0	0	0%
Omitting Zhou, D. 2022	1.38	[0.68; 2.80]	0.3661	0	0	0%
Omitting Forrest, J.K. 2020	1.61	[0.79; 3.26]	0.1877	0	0	0%
Omitting Liu, X.B. 2015	1.37	[0.68; 2.77]	0.3824	0	0	0%
Omitting Michel, J.M. 2021	1.46	[0.73; 2.92]	0.2798	0	0	0%
Omitting Pineda, A.M. 2020	1.46	[0.73; 2.92]	0.2822	0	0	0%
Omitting Song, G.Y. 2018	1.34	[0.66; 2.71]	0.4219	0	0	0%
**Random effects model**	**1.44**	**[0.73; 2.87]**	**0.2947**	**0**	**0**	**0%**

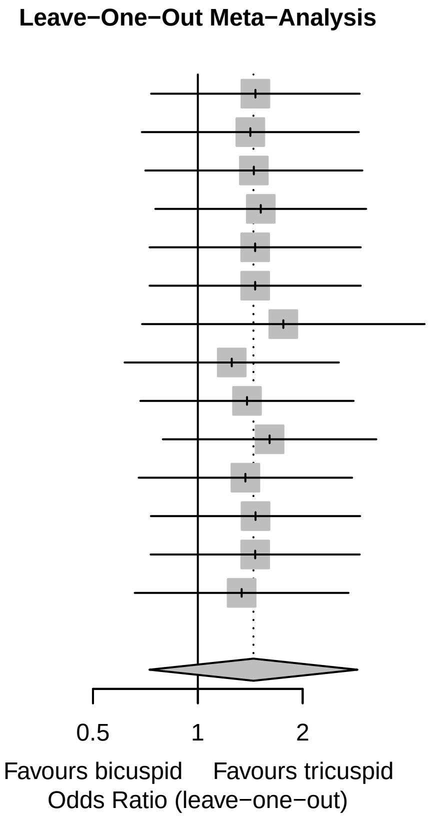

(E) Sensitivity Analysis for Coronary obstruction

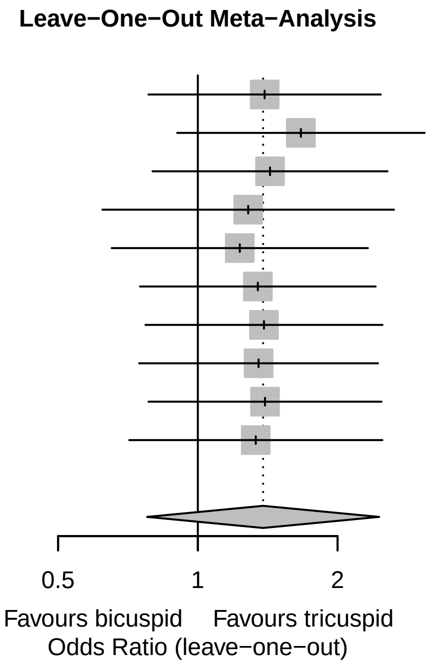

Study omitted	OR	95%−CI	P−value	Tau2	Tau	I2
Omitting Costopoulos, C. 2014	1.39	[0.78; 2.48]	0.2597	0	0	0%
Omitting Dai, H. 2023	1.67	[0.90; 3.08]	0.1026	0	0	0%
Omitting Fu, B. 2020	1.43	[0.80; 2.56]	0.2285	0	0	0%
Omitting Makkar, R.R. 2019	1.28	[0.62; 2.65]	0.4984	0	0	0%
Omitting Makkar, R.R. 2021	1.23	[0.65; 2.32]	0.5210	0	0	0%
Omitting Mangieri, A. 2018	1.35	[0.75; 2.42]	0.3183	0	0	0%
Omitting Zhou, D. 2022	1.39	[0.77; 2.50]	0.2742	0	0	0%
Omitting Forrest, J.K. 2020	1.35	[0.75; 2.44]	0.3189	0	0	0%
Omitting Michel, J.M. 2021	1.40	[0.78; 2.49]	0.2584	0	0	0%
Omitting Yoon, S.H. 2017	1.33	[0.71; 2.50]	0.3696	0	0	0%
**Random effects model**	**1.38**	**[0.78; 2.46]**	**0.2699**	**0**	**0**	**0%**

(F) Sensitivity Analysis for Moderate/severe PVL

Study omitted	OR	95%−CI	P−value	Tau2	Tau	I2
Omitting Fu, B. 2020	1.38	[1.22; 1.57]	< 0.0001	0	0	0%
Omitting Hong, N. 2023	1.39	[1.22; 1.58]	< 0.0001	0	0	0%
Omitting Medranda, G.A. 2021	1.39	[1.22; 1.58]	< 0.0001	0	0	0%
Omitting Zhou, D. 2022	1.41	[1.24; 1.61]	< 0.0001	0	0	0%
Omitting Chodór, P.A. 2021	1.39	[1.22; 1.57]	< 0.0001	0	0	0%
Omitting De Biase, C. 2018	1.39	[1.22; 1.58]	< 0.0001	0	0	0%
Omitting Kim, W.K. 2021	1.40	[1.23; 1.59]	< 0.0001	0	0	0%
Omitting Michel, J.M. 2021	1.39	[1.22; 1.58]	< 0.0001	0	0	0%
Omitting Song, G.Y. 2018	1.38	[1.22; 1.57]	< 0.0001	0	0	0%
Omitting Yoon, S.H. 2017	1.24	[0.92; 1.66]	0.1518	0.0315	0.1776	0%
Omitting Arai, T. 2017	1.39	[1.22; 1.58]	< 0.0001	0	0	0%
Omitting Halim, S. A. 2020	1.21	[0.84; 1.76]	0.3059	0.0432	0.2078	0%
**Random effects model**	**1.39**	**[1.22; 1.58]**	**< 0.0001**	**0**	**0**	**0%**

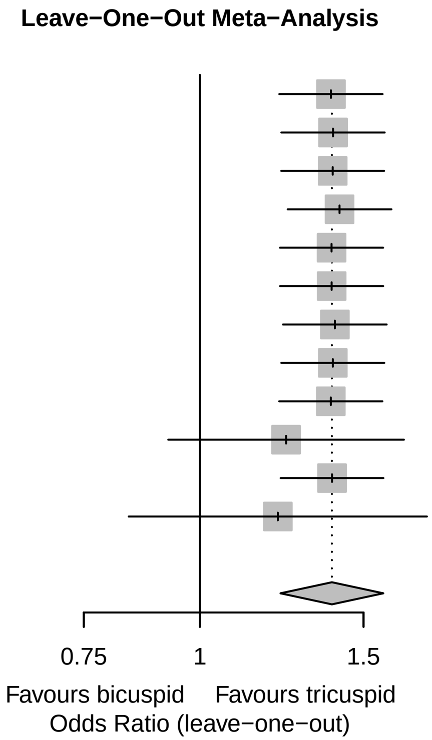

(G) Sensitivity Analysis for Major vascular complications

Study omitted	OR	95%−CI	P−value	Tau2	Tau	I2
Omitting Costopoulos, C. 2014	0.95	[0.72; 1.25]	0.7036	0	0	0%
Omitting Fu, B. 2020	0.92	[0.70; 1.21]	0.5305	0	0	0%
Omitting Hong, N. 2023	0.95	[0.71; 1.25]	0.6916	0	0	0%
Omitting Kochman, J. 2014	0.95	[0.73; 1.25]	0.7234	0	0	0%
Omitting Liu, K. 2024	0.95	[0.73; 1.25]	0.7349	0	0	0%
Omitting Magyari, B. 2024	0.96	[0.73; 1.26]	0.7624	0	0	0%
Omitting Makkar, R.R. 2019	0.94	[0.69; 1.28]	0.7119	0	0	0%
Omitting Makkar, R.R. 2021	0.96	[0.71; 1.29]	0.7805	0	0	0%
Omitting Forrest, J.K. 2020	0.94	[0.71; 1.25]	0.6766	0	0	0%
Omitting Kim, W.K. 2021	0.95	[0.70; 1.30]	0.7662	0	0	0%
Omitting Liu, X.B. 2015	0.94	[0.71; 1.23]	0.6353	0	0	0%
Omitting Michel, J.M. 2021	0.97	[0.73; 1.29]	0.8280	0	0	0%
Omitting Pineda, A.M. 2020	0.94	[0.71; 1.23]	0.6434	0	0	0%
Omitting Song, G.Y. 2018	0.96	[0.73; 1.26]	0.7608	0	0	0%
Omitting Arai, T. 2017	0.93	[0.71; 1.23]	0.6291	0	0	0%
**Random effects model**	**0.95**	**[0.72; 1.24]**	**0.6930**	**0**	**0**	**0%**

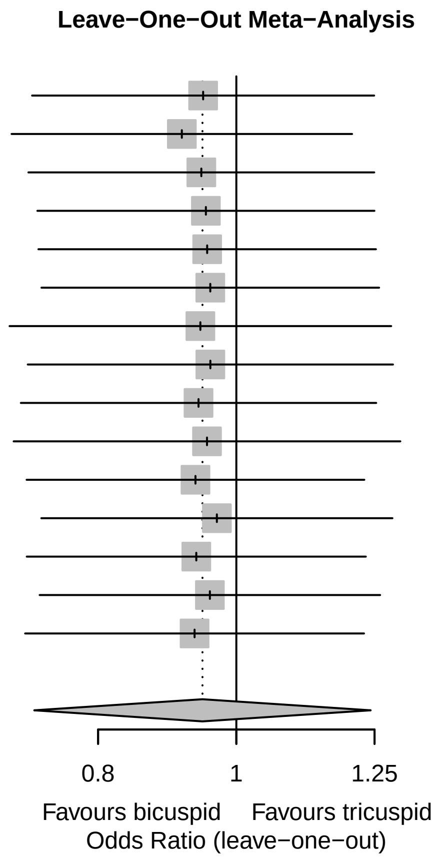

(H) Sensitivity Analysis for 30-day All-cause mortality

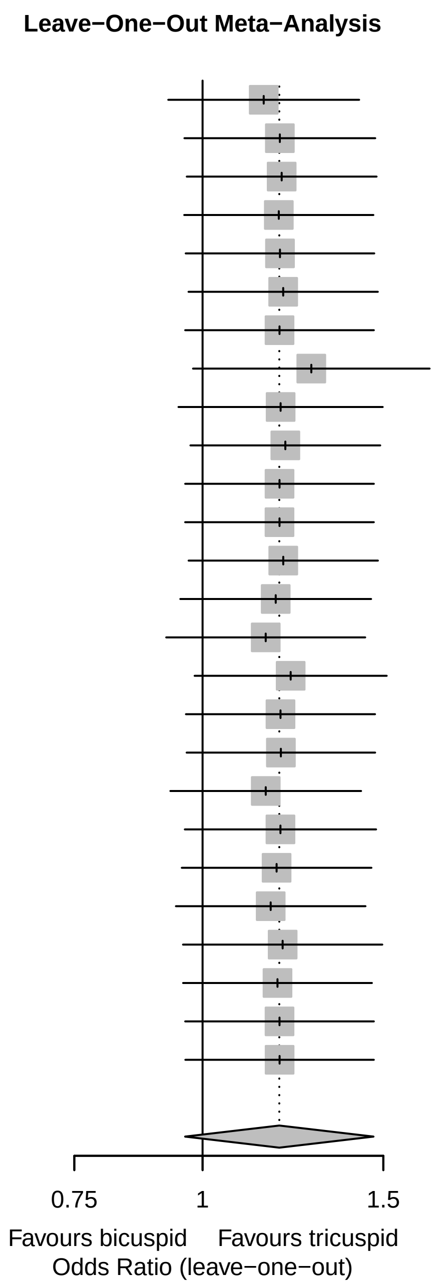

Study omitted	OR	95%−CI	P−value	Tau2	Tau	I2
Omitting Costopoulos, C. 2014	1.15	[0.93; 1.42]	0.2091	0	0	0%
Omitting Dai, H. 2023	1.19	[0.96; 1.47]	0.1125	0	0	0%
Omitting Fu, B. 2020	1.19	[0.97; 1.48]	0.1023	0	0	0%
Omitting Hong, N. 2023	1.19	[0.96; 1.47]	0.1141	0	0	0%
Omitting Kawamori, H. 2018	1.19	[0.96; 1.47]	0.1076	0	0	0%
Omitting Kochman, J. 2014	1.20	[0.97; 1.48]	0.0950	0	0	0%
Omitting Magyari, B. 2024	1.19	[0.96; 1.47]	0.1098	0	0	0%
Omitting Makkar, R.R. 2019	1.28	[0.98; 1.66]	0.0716	0	0	0%
Omitting Makkar, R.R. 2021	1.19	[0.95; 1.50]	0.1342	0	0	0%
Omitting Mangieri, A. 2018	1.20	[0.97; 1.49]	0.0874	0	0	0%
Omitting Medranda, G.A. 2021	1.19	[0.96; 1.47]	0.1098	0	0	0%
Omitting Xu, Q. 2021	1.19	[0.96; 1.47]	0.1099	0	0	0%
Omitting Chodór, P.A. 2021	1.20	[0.97; 1.48]	0.0947	0	0	0%
Omitting De Biase, C. 2018	1.18	[0.95; 1.46]	0.1328	0	0	0%
Omitting Forrest, J.K. 2020	1.15	[0.92; 1.44]	0.2135	0	0	0%
Omitting Kim, W.K. 2021	1.22	[0.98; 1.51]	0.0722	0	0	0%
Omitting Liu, X.B. 2015	1.19	[0.96; 1.47]	0.1062	0	0	0%
Omitting Michel, J.M. 2021	1.19	[0.96; 1.47]	0.1034	0	0	0%
Omitting Pineda, A.M. 2020	1.15	[0.93; 1.43]	0.1941	0	0	0%
Omitting Sannino, A. 2017	1.19	[0.96; 1.48]	0.1105	0	0	0%
Omitting Song, G.Y. 2018	1.18	[0.95; 1.46]	0.1260	0	0	0%
Omitting Tung, M. 2018	1.17	[0.94; 1.44]	0.1590	0	0	0%
Omitting Yoon, S.H. 2017	1.20	[0.96; 1.50]	0.1152	0	0	0%
Omitting Deeb, G.M. 2022	1.18	[0.96; 1.46]	0.1198	0	0	0%
Omitting Williams, M.R. 2022	1.19	[0.96; 1.47]	0.1098	0	0	0%
Omitting Arai, T. 2017	1.19	[0.96; 1.47]	0.1091	0	0	0%
**Random effects model**	**1.19**	**[0.96; 1.47]**	**0.1104**	**0**	**0**	**0%**

(I) Sensitivity Analysis for 30-day Cardiovascular mortality

Study omitted	OR	95%−CI	P−value	Tau2	Tau	I2
Omitting Costopoulos, C. 2014	1.37	[0.71; 2.62]	0.3496	0	0	0%
Omitting Dai, H. 2023	1.67	[0.87; 3.23]	0.1244	0	0	0%
Omitting Fu, B. 2020	1.74	[0.91; 3.30]	0.0924	0	0	0%
Omitting Magyari, B. 2024	1.61	[0.87; 2.96]	0.1270	0	0	0%
Omitting Xiong, T.Y. 2018	1.66	[0.88; 3.14]	0.1185	0	0	0%
Omitting Liu, X.B. 2015	1.66	[0.90; 3.05]	0.1050	0	0	0%
Omitting Pineda, A.M. 2020	1.46	[0.76; 2.79]	0.2579	0	0	0%
Omitting Sannino, A. 2017	1.62	[0.81; 3.22]	0.1687	0	0	0%
Omitting Deeb, G.M. 2022	1.55	[0.84; 2.87]	0.1596	0	0	0%
**Random effects model**	**1.59**	**[0.87; 2.90]**	**0.1315**	**0**	**0**	**0%**

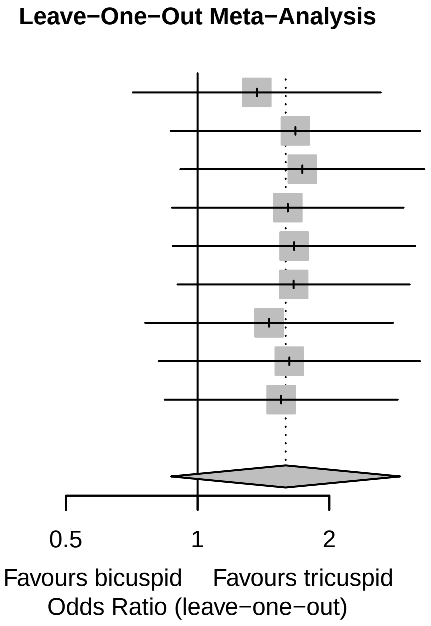

(J) Sensitivity Analysis for 1year All-cause mortality

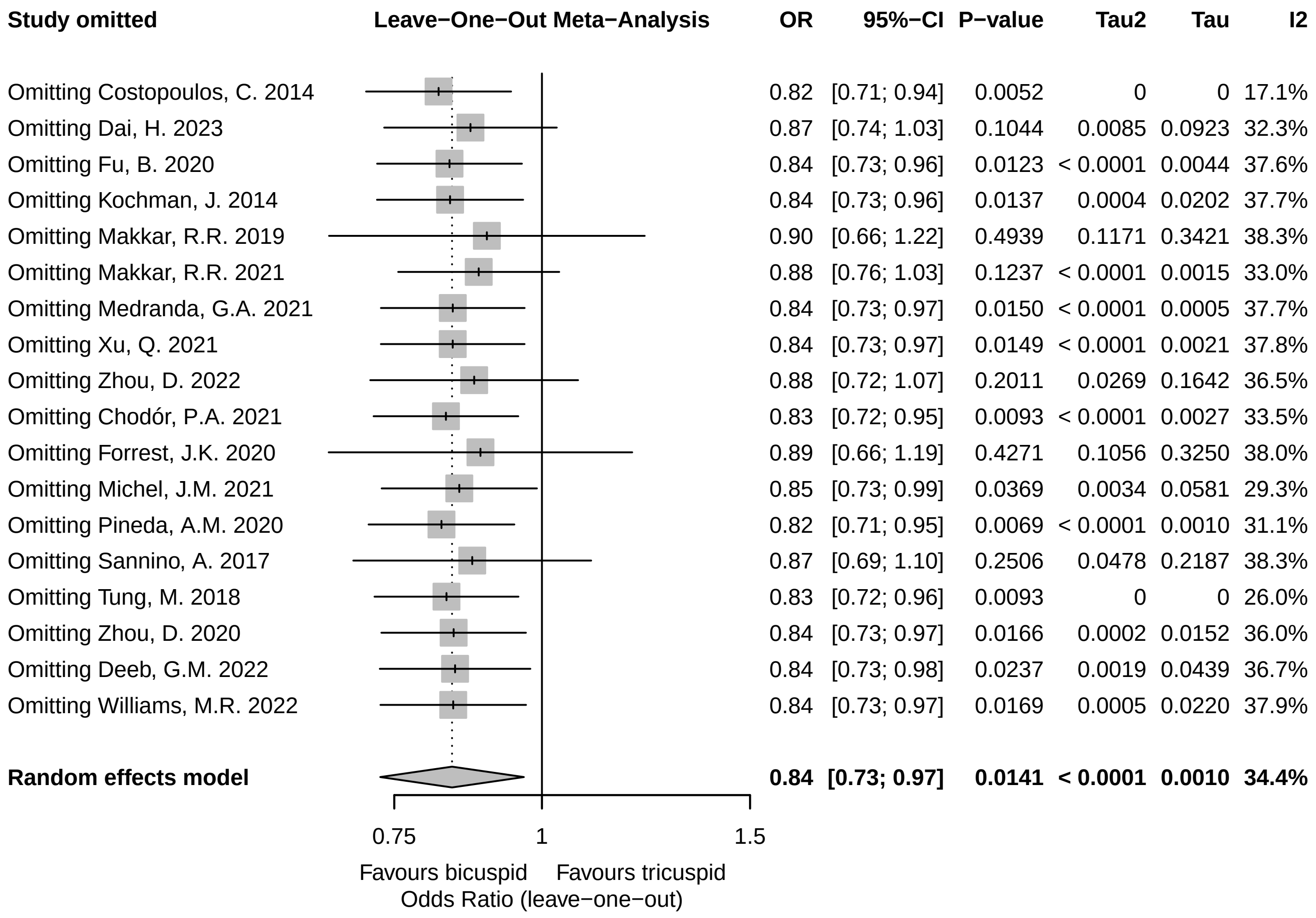

(K) Sensitivity Analysis for 1year Cardiovascular mortality

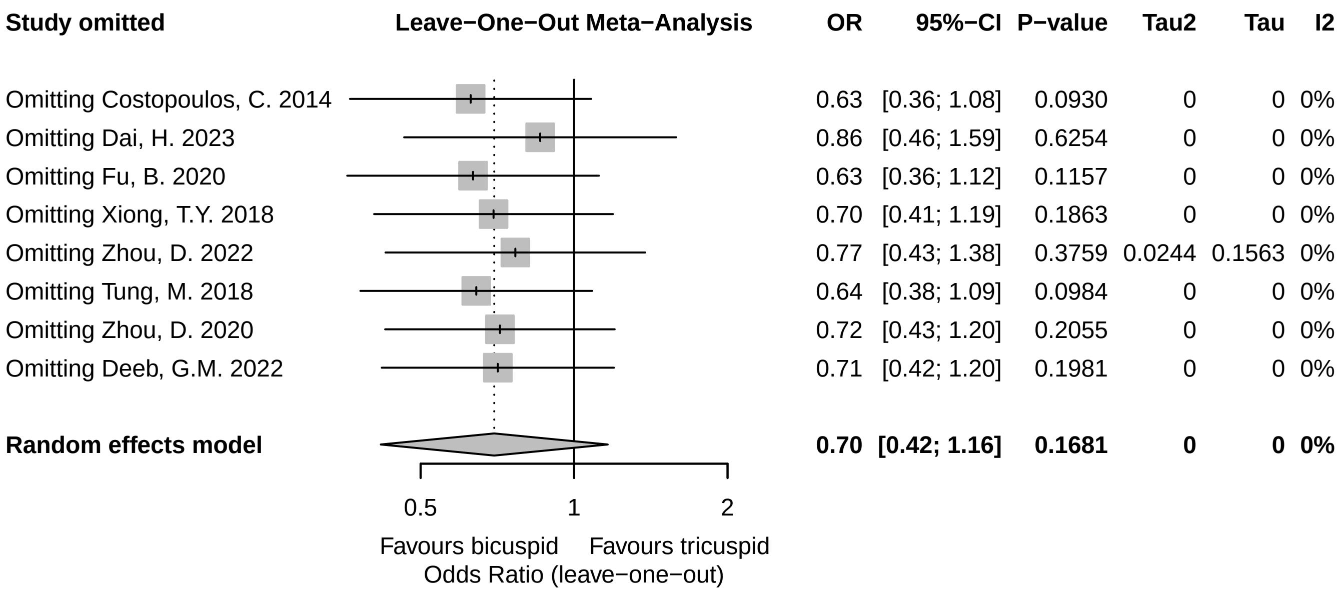

Supplementary Figure 4

Supplementary Figure 4. Sensitivity analyses of clinical endpoints excluding studies with a Newcastle-Ottawa Scale (NOS) score <7.

(A) Sensitivity Analysis for Device success with NOS

Leave−One−Out Meta−Analysis

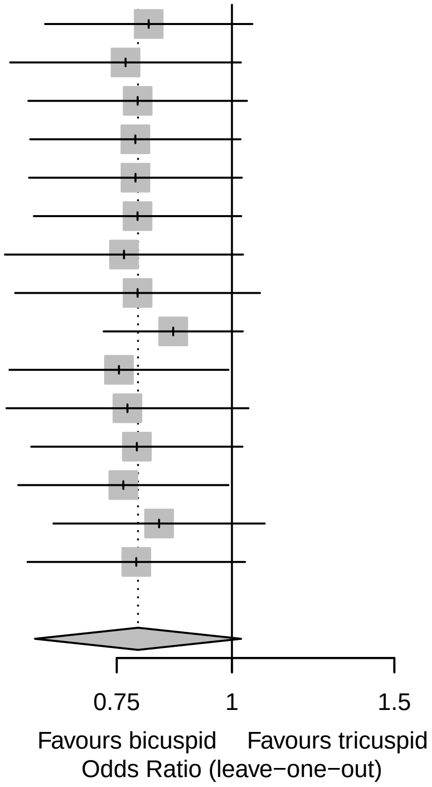

Study omitted	OR	95%−CI	P−value	Tau2	Tau	I2
Omitting Costopoulos, C. 2014	0.81	[0.63; 1.05]	0.1155	0.1039	0.3224	46.5%
Omitting Dai, H. 2023	0.77	[0.57; 1.02]	0.0707	0.1304	0.3611	48.5%
Omitting Fu, B. 2020	0.79	[0.60; 1.04]	0.0909	0.1213	0.3482	49.9%
Omitting Kawamori, H. 2018	0.79	[0.60; 1.02]	0.0718	0.1121	0.3348	49.7%
Omitting Kochman, J. 2014	0.79	[0.60; 1.03]	0.0755	0.1148	0.3388	49.8%
Omitting Magyari, B. 2024	0.79	[0.61; 1.02]	0.0744	0.1089	0.3299	49.9%
Omitting Makkar, R.R. 2019	0.76	[0.57; 1.03]	0.0756	0.1368	0.3699	46.6%
Omitting Makkar, R.R. 2021	0.79	[0.58; 1.07]	0.1310	0.1495	0.3866	49.2%
Omitting De Biase, C. 2018	0.86	[0.73; 1.03]	0.0982	0.0206	0.1436	0%
Omitting Forrest, J.K. 2020	0.75	[0.57; 0.99]	0.0432	0.1090	0.3301	44.5%
Omitting Kim, W.K. 2021	0.77	[0.57; 1.04]	0.0906	0.1441	0.3797	48.7%
Omitting Liu, X.B. 2015	0.79	[0.61; 1.03]	0.0777	0.1132	0.3364	49.9%
Omitting Michel, J.M. 2021	0.76	[0.59; 0.99]	0.0429	0.1054	0.3246	46.0%
Omitting Yoon, S.H. 2017	0.83	[0.64; 1.09]	0.1765	0.0945	0.3075	38.1%
Omitting Deeb, G.M. 2022	0.79	[0.60; 1.03]	0.0848	0.1201	0.3466	49.9%
**Random effects model**	**0.79**	**[0.61; 1.02]**	**0.0740**	**0.1074**	**0.3277**	**46.1%**

(B) Sensitivity Analysis for In-hospital mortality with NOS

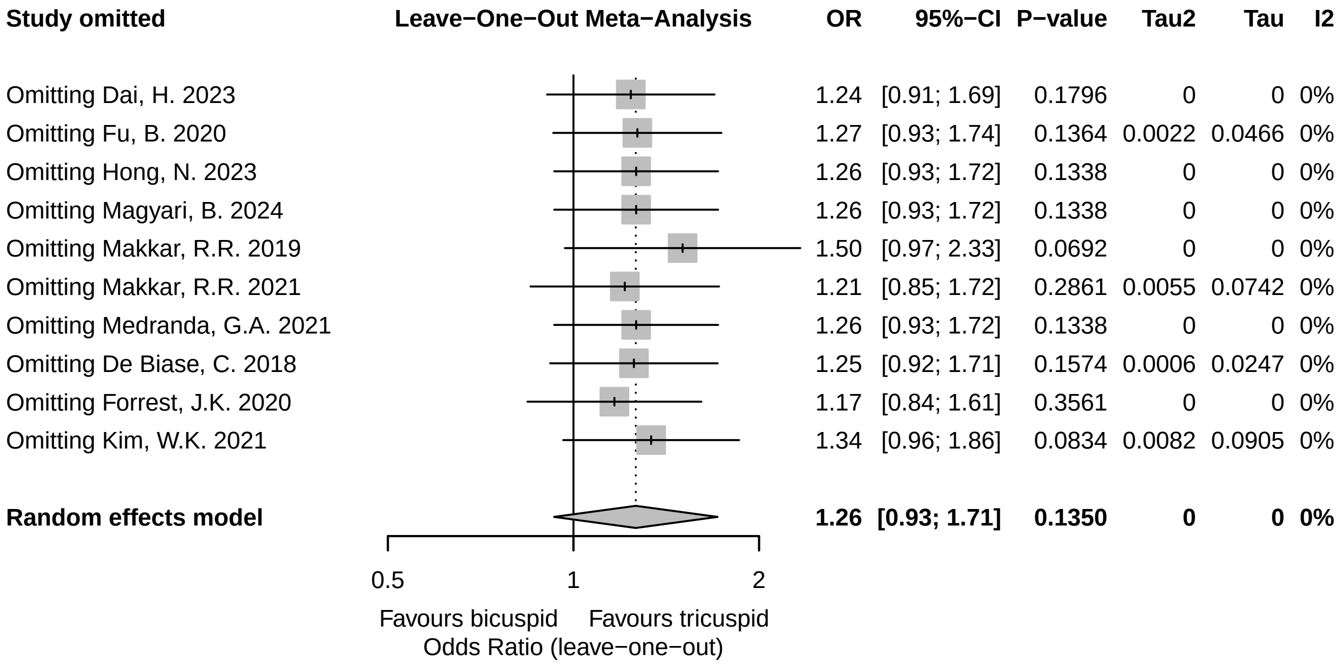

(C) Sensitivity Analysis for Bleeding life-threatening with NOS

Study omitted	OR	95%−CI	P−value	Tau2	Tau	I2
Omitting Costopoulos, C. 2014	1.00	[0.61; 1.64]	0.9962	0	0	0%
Omitting Kochman, J. 2014	0.97	[0.58; 1.63]	0.9215	0	0	0%
Omitting Liu, K. 2024	0.94	[0.58; 1.52]	0.7915	0	0	0%
Omitting Makkar, R.R. 2019	0.94	[0.58; 1.52]	0.7915	0	0	0%
Omitting Makkar, R.R. 2021	0.94	[0.58; 1.52]	0.7915	0	0	0%
Omitting Kim, W.K. 2021	0.80	[0.33; 1.94]	0.6285	0	0	0%
Omitting Liu, X.B. 2015	0.95	[0.59; 1.55]	0.8474	0	0	0%
Omitting Pineda, A.M. 2020	0.87	[0.54; 1.42]	0.5791	0	0	0%
Omitting Sannino, A. 2017	0.95	[0.59; 1.53]	0.8214	0	0	0%
Omitting Song, G.Y. 2018	0.94	[0.58; 1.52]	0.7915	0	0	0%
**Random effects model**	**0.94**	**[0.58; 1.51]**	**0.7930**	**0**	**0**	**0%**

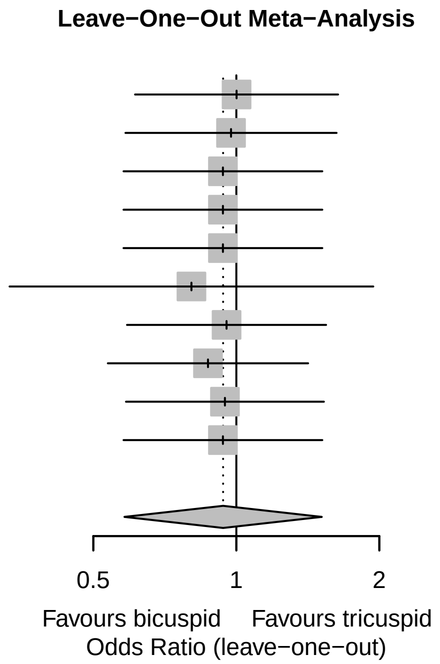

(D) Sensitivity Analysis for Moderate/severe PVL with NOS

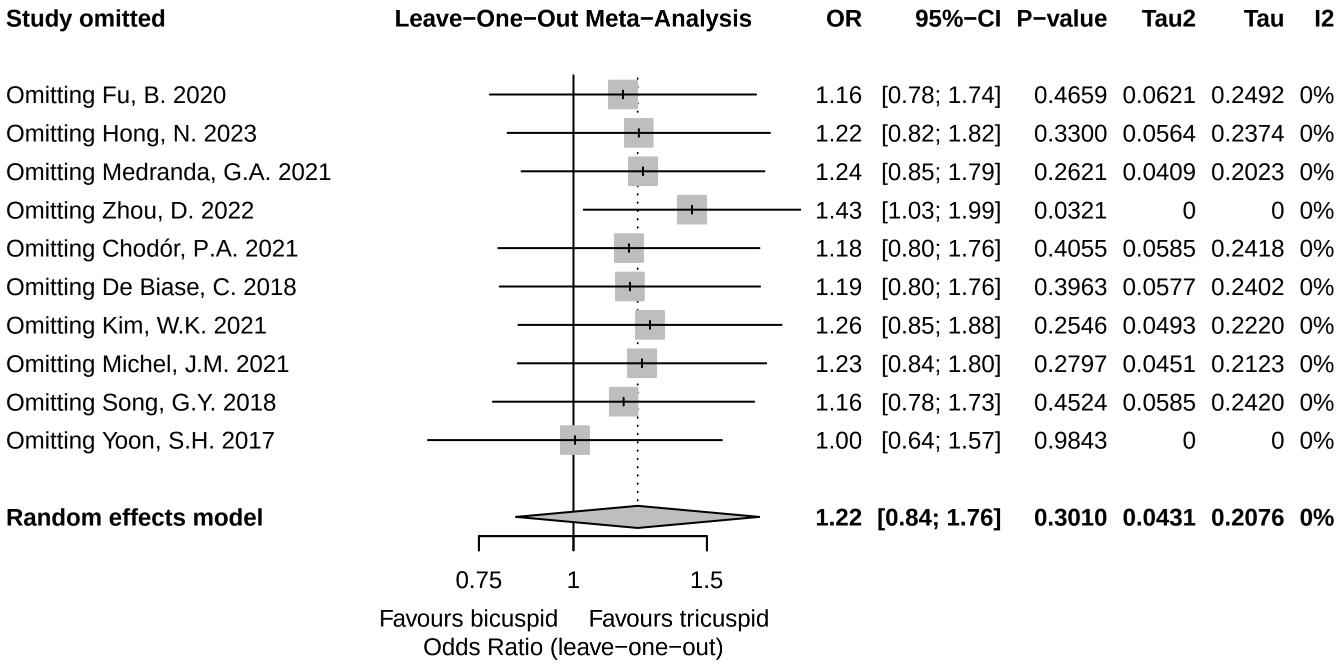

(E) Sensitivity Analysis for Major vascular complications with NOS

Study omitted	OR	95%−CI	P−value	Tau2	Tau	I2
Omitting Costopoulos, C. 2014	0.94	[0.71; 1.24]	0.6383	0	0	0%
Omitting Fu, B. 2020	0.90	[0.68; 1.19]	0.4725	0	0	0%
Omitting Hong, N. 2023	0.93	[0.70; 1.24]	0.6259	0	0	0%
Omitting Kochman, J. 2014	0.94	[0.71; 1.24]	0.6585	0	0	0%
Omitting Liu, K. 2024	0.94	[0.72; 1.24]	0.6696	0	0	0%
Omitting Magyari, B. 2024	0.95	[0.72; 1.25]	0.6964	0	0	0%
Omitting Makkar, R.R. 2019	0.93	[0.68; 1.27]	0.6385	0	0	0%
Omitting Makkar, R.R. 2021	0.94	[0.70; 1.27]	0.7086	0	0	0%
Omitting Forrest, J.K. 2020	0.93	[0.69; 1.24]	0.6095	0	0	0%
Omitting Kim, W.K. 2021	0.94	[0.68; 1.29]	0.6903	0	0	0%
Omitting Liu, X.B. 2015	0.92	[0.70; 1.22]	0.5731	0	0	0%
Omitting Michel, J.M. 2021	0.96	[0.72; 1.27]	0.7581	0	0	0%
Omitting Pineda, A.M. 2020	0.93	[0.70; 1.22]	0.5807	0	0	0%
Omitting Song, G.Y. 2018	0.95	[0.72; 1.25]	0.6943	0	0	0%
**Random effects model**	**0.93**	**[0.71; 1.23]**	**0.6291**	**0**	**0**	**0%**

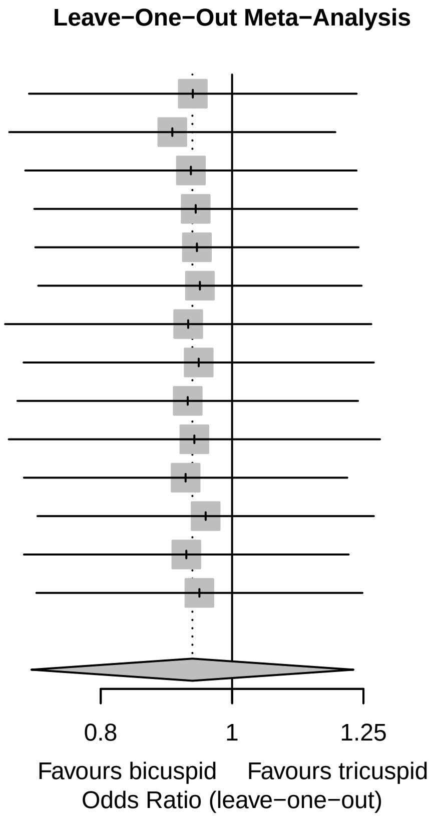

(F) Sensitivity Analysis for 30-day All-cause mortality with NOS

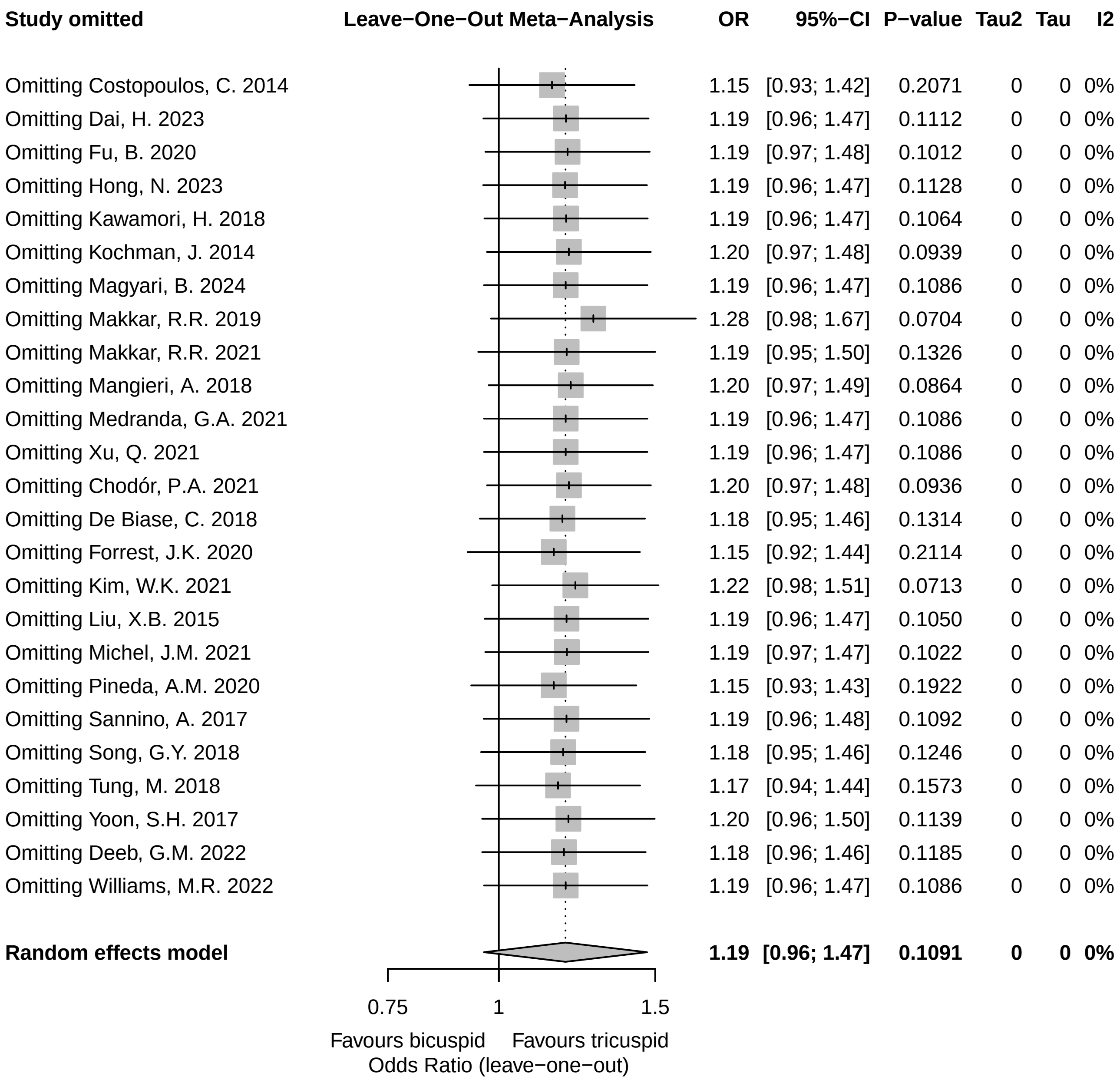

Supplement: Supplementary file 1 [file 2153-8174-27-7-49401-s1.zip › Supplementary Material.pdf]
